# Alignment of virus-host protein-protein interaction networks by integer linear programming: SARS-CoV-2

**DOI:** 10.1371/journal.pone.0236304

**Published:** 2020-12-07

**Authors:** Mercè Llabrés, Gabriel Valiente

**Affiliations:** 1 Department of Mathematics and Computer Science, University of the Balearic Islands, Palma de Mallorca, Spain; 2 Algorithms, Bioinformatics, Complexity and Formal Methods Research Group, Technical University of Catalonia, Barcelona, Spain; University of Pittsburgh, UNITED STATES

## Abstract

**Motivation:**

Beside socio-economic issues, coronavirus pandemic COVID-19, the infectious disease caused by the newly discovered coronavirus SARS-CoV-2, has caused a deep impact in the scientific community, that has considerably increased its effort to discover the infection strategies of the new virus. Among the extensive and crucial research that has been carried out in the last months, the analysis of the virus-host relationship plays an important role in drug discovery. Virus-host protein-protein interactions are the active agents in virus replication, and the analysis of virus-host protein-protein interaction networks is fundamental to the study of the virus-host relationship.

**Results:**

We have adapted and implemented a recent integer linear programming model for protein-protein interaction network alignment to virus-host networks, and obtained a consensus alignment of the SARS-CoV-1 and SARS-CoV-2 virus-host protein-protein interaction networks. Despite the lack of shared human proteins in these virus-host networks, and the low number of preserved virus-host interactions, the consensus alignment revealed aligned human proteins that share a function related to viral infection, as well as human proteins of high functional similarity that interact with SARS-CoV-1 and SARS-CoV-2 proteins, whose alignment would preserve these virus-host interactions.

## 1 Introduction

The present outbreak of a coronavirus-associated acute respiratory disease, the COVID-19 pandemic, has forced the scientific community to rapidly analyze the virus-host relationships of the new coronavirus (SARS-CoV-2) human infection. Thus, in less than a month, several databases as [1–3] have been created to collect all SARS-CoV-2 and COVID-19 information, and the SARS-CoV-2-human protein-protein interaction network was built [4]. As stated in [5],

The *Coronaviridae Study Group* (CSG) of the International Committee on Taxonomy of Viruses […] has assessed the placement of the human pathogen, tentatively named 2019-nCoV, within the *Coronaviridae*. Based on phylogeny, taxonomy and established practice, the CSG recognizes this virus as forming a sister clade to the prototype human and bat severe acute respiratory syndrome coronaviruses (SARS-CoVs) of the species *Severe acute respiratory syndrome-related coronavirus*, and designates it as SARS-CoV-2.

Therefore, the closest known human pathogen to SARS-CoV-2 is the coronavirus SARS-CoV that appeared in 2003 [6], also called SARS-CoV-1.

Understanding the mechanism of the SARS-CoV-2 infection is a crucial step towards the discovery of antiviral drugs and vaccines. The *modus operandi* of every viral infection is through the interaction between viral proteins and host proteins, in order to use the host cells to replicate. In this line of research, virus-host protein-protein interaction networks, a particular form of protein-protein interaction networks, have become appropriate to analyze virus-host relationships, and information on well-known and studied virus-host protein-protein interaction networks can be carried over to new ones by way of protein-protein interaction network comparison and alignment. See [7, 8] for comprehensive reviews.

The general problem of protein-protein interaction network alignment has been explored in the last two decades, and several tools have been already proposed and implemented [9–14]. However, the particular case of virus-host protein-protein interaction network alignment problem has not been fully studied yet.

We have recently developed a compact reformulation of a quadratic programming model for the protein-protein interaction network alignment problem as an integer linear program, which has been proven to be suitable for the alignment of virus-host protein-protein interaction networks [15]. Our proposed model can be solved using state-of-the-art mathematical modeling software such as AMPL [16] and integer linear programming software tools such as IBM ILOG CPLEX Optimization Studio and Gurobi Optimizer. In this work, we adapt and implement a modification of the aforementioned alignment method to align the virus-host protein-protein interaction networks of SARS-CoV-1 and SARS-CoV-2, in order to elucidate information on the infection mechanism of SARS-CoV-2 based on current knowledge on the infection mechanism of SARS-CoV-1.

## 2 Methods

In the integer linear programming formulation of the protein-protein interaction network alignment problem, described in [15], a virus-host protein-protein interaction network is represented by an undirected bipartite graph *G* = (*U*, *V*, *E*), with a node *u* ∈ *U* for each virus protein, a node *v* ∈ *V* for each host protein, and an edge {*u*, *v*} ∈ *E* for each virus-host protein-protein interaction. Notice that these bipartite graphs need not be connected.

Let *G* = (*U*, *V*, *E*) and *G*′ = (*U*′, *V*′, *E*′) be the two virus-host protein-protein interaction networks to be aligned, and let *A* = (*a*_*ij*_) and *B* = (*b*_*k*ℓ_) be their weighted adjacency matrices, where the weight of an entry *a*_*ij*_ ∈ [0, 1] is the confidence score of the interaction {*i*, *j*} ∈ *E*, and the weight of an entry *b*_*k*ℓ_ ∈ [0, 1] is the confidence score of the interaction {*k*, ℓ} ∈ *E*′. Let also *S* = (*s*_*ik*_) be a similarity matrix between the nodes of the two networks, with each *s*_*ik*_ ∈ [0, 1] the similarity score of *i* ∈ *U*∪*V* and *k* ∈ *U*′ ∪ *V*′.

Let us define a binary variable *x*_*ik*_ for each *i* ∈ *U* ∪ *V* and each *k* ∈ *U*′ ∪ *V*′, where *x*_*ik*_ = 1 if node *i* of the first network is aligned with node *k* of the second network, and *x*_*ik*_ = 0 otherwise. Then, an alignment of two virus-host protein-protein interaction networks *G* = (*U*, *V*, *E*) and *G*′ = (*U*′, *V*′, *E*′) is represented by the binary matrix *X* = (*x*_*ik*_). Let us also define an integer variable *y*_*ik*_ for each *i* ∈ *U*∪*V* and each *k* ∈ *U*′ ∪ *V*′, where each integer variable *y*_*ik*_ is intended to represent

$$\begin{array}{c} y_{ik}=x_{ik}\sum_{j\in U\cup V}\sum_{\ell \in U^{\prime }\cup V^{\prime }}a_{ij}b_{k\ell }x_{j\ell } \end{array}$$

for *i* ∈ *U* ∪ *V* and *k* ∈ *U*′ ∪ *V*′. In this way, if *x*_*ik*_ = 0, *y*_*ik*_ = 0, and if *x*_*ik*_ = 1, *y*_*ik*_ is the weight of those edges incident to node *i* in *G* that are preserved by the alignment.

Then, the goal of the integer linear programming model is to maximize

$$\begin{array}{c} \lambda \sum_{i\in U\cup V}\sum_{k\in U^{\prime }\cup V^{\prime }}s_{ik}\;x_{ik}+\left(1-\lambda \right)\sum_{i\in U\cup V}\sum_{k\in U^{\prime }\cup V^{\prime }}y_{ik} \end{array}$$

subject to the constraints

- $\sum_{k\in U^{\prime }\cup V^{\prime }}x_{ik}⩽1,\;i\in U\cup V$
- $\sum_{i\in U\cup V}x_{ik}⩽1,\;k\in U^{\prime }\cup V^{\prime }$
- $0⩽y_{ik}⩽x_{ik}\sum_{j\in U\cup V}\sum_{l\in U^{\prime }\cup V^{\prime }}a_{ij}b_{kl},\;i\in U\cup V,\;k\in U^{\prime }\cup V^{\prime }$
- $y_{ik}⩽\sum_{j\in U\cup V}\sum_{l\in U^{\prime }\cup V^{\prime }}a_{ij}b_{kl}x_{jl}\$,\;\$i\in U\cup V,\;k\in U^{\prime }\cup V^{\prime }$

where *λ* is a parameter, with 0 ≤ *λ* ≤ 1, to control the balance between protein similarity scores and protein-protein interaction weights: only node scores are considered when *λ* = 1, and only edge scores are taken into account when *λ* = 0.

It is easy to see that this integer linear programming formulation is equivalent to the integer quadratic programming formulation of the network alignment problem given in [9]. In fact, the previous constraints entail

$$\begin{array}{c} \sum_{i\in U\cup V}\sum_{k\in U^{\prime }\cup V^{\prime }}y_{ik}=\sum_{i\in U\cup V}\sum_{k\in U^{\prime }\cup V^{\prime }}\sum_{j\in U\cup V}\sum_{\ell \in U^{\prime }\cup V^{\prime }}a_{ij}\;b_{k\ell }\;x_{ik}\;x_{j\ell } \end{array}$$

The objective function comes from the PathBLAST [11] idea that protein-protein network alignment be based on a log-probability-like criterion, with matching terms corresponding to both proteins and interactions [9]. The first sum in the objective function,

$$\begin{array}{c} \sum_{i\in U\cup V}\sum_{k\in U^{\prime }\cup V^{\prime }}s_{ik}\;x_{ik}, \end{array}$$

represents the global similarity of the aligned proteins, while the second sum,

$$\begin{array}{c} \sum_{i\in U\cup V}\sum_{k\in U^{\prime }\cup V^{\prime }}\sum_{j\in U\cup V}\sum_{\ell \in U^{\prime }\cup V^{\prime }}a_{ij}\;b_{k\ell }\;x_{ik}\;x_{j\ell }, \end{array}$$

represents the weight of those edges that are preserved by the alignment; that is, those pairs of edges (*i*, *j*)∈*E* and (*k*, ℓ)∈*E*′ such that node *i* is aligned with node *k* and node *j* is aligned with node ℓ.

Let *m* = |*U*| + |*V*| and *n* = |*U*′| + |*V*′|. The resulting integer linear programming formulation of the virus-host protein-protein network alignment problem has *O*(*mn*) binary variables, integer variables, and constraints.

## 3 Results and discussion

There are 130 interactions between 29 SARS-CoV-1 proteins and 109 human proteins in the March 2020 release of the VirHostNet database [1], as well as 332 interactions between 26 SARS-CoV-2 proteins and 332 human proteins from [4] in release 4.2.13 of the IntAct database [2]. Thus, the SARS-CoV-1-Human network has 138 nodes and 130 edges, while the SARS-CoV-2-Human network has 358 nodes and 332 edges. Notice that only 6 of these 109 and 332 human proteins (P27448, Q5JRX3, Q7KZI7, Q9BW92, Q9H4F8, and Q9Y6E2) interact with both SARS-CoV-1 and SARS-CoV-2 proteins. Notice also that we have excluded any interactions among the 109 human proteins in the SARS-CoV-1-Human network, as well as any interactions among the 332 human proteins in the SARS-CoV-2-Human network. These host-host interactions do not contribute to improving the quality of the virus-host protein-protein network alignment, they rather introduce noise and, in fact, the inclusion of both virus-host and host-host interactions in the SARS-CoV-1-Human and SARS-CoV-2-Human networks results in the alignment of four of the SARS-CoV-1 proteins (nsp7, nsp9, nsp12, and orf7a) with host proteins (P61019, P06280, Q9Y375, and Q9Y5J7, respectively) instead of SARS-CoV-2 proteins. The SARS-CoV-1-Human and SARS-CoV-2-Human virus-host protein-protein interaction networks have 11 and 26 connected components, respectively.

We have obtained the amino acid sequences for the SARS-CoV-1 and human proteins from UniProt/SwissProt (122 sequences), UniProt/TrEMBL (2 sequences), and NCBI RefSeq (14 sequences), and for the SARS-CoV-2 and human proteins from UniProt/ SwissProt (332 sequences) and from the Supplementary material in [4] (26 sequences). We have taken the global alignment score between the amino acid sequences of two proteins, computed by dynamic programming with the algorithm of [17] as implemented in BioPython [18], with a gap opening penalty of −7 and a gap extension penalty of −1, and normalized to [0, 1], as the similarity score between the proteins. In the protein sequences of P07203 and Q9BQE4, we substituted C (cysteine) for the rare amino acid U (selenocysteine), which appears only once in the protein sequences of P07203 and Q9BQE4 over a dataset of 310,717 amino acids in 496 viral and human protein sequences, in order to compute global sequence alignments using the BLOSUM62 amino acid substitution matrix, which does not cover selenocysteine. The corresponding integer linear programming problem instance has 83,628 variables, half of which are binary, and 84,069 constraints.

The alignment of the virus-host protein-protein interaction networks of SARS-CoV-1 and SARS-CoV-2 was computed with AMPL version 2018.10.22 [16] and Gurobi Optimizer version 8.1.0, using a personal computer with an Intel Core i7-8550U quad-core processor at 1.80 GHz and 32 GB of memory running Ubuntu 18.04 LTS. The optimal alignment was found in 517.35 seconds of AMPL time, plus 3.16697 seconds of solver time, for SARS-CoV-1 to SARS-CoV-2, and in 538.112 seconds of AMPL time, plus 3.45882 seconds of solver time, for SARS-CoV-2 to SARS-CoV-1. We set *λ* = 0.5 in both cases, and took the consensus between them as the alignment of the two virus-host protein-protein interaction networks.

Protein similarity can be assessed by comparing the annotated Gene Ontology (GO) terms for the proteins along three classifications: the molecular function ontology (MFO), the biological process ontology (BPO), and the cellular component ontology (CCO). We considered the host proteins that interact with the viral proteins in the consensus alignment, for each of the two networks. For these human proteins we obtained their GO term annotations using GOnet [19], and measured the functional similarity between aligned human proteins using GOGO [20], which computes the average best semantic similarity between the GO term annotations for the proteins based on their shortest paths in the GO classifications. Tables 1 (structural proteins), 2–4 (non-structural proteins), and 5 (accessory proteins) show the alignment of viral proteins in the consensus alignment, along with the alignment of the human proteins they interact with, their MFO score, their BPO score, and their CCO score.

**Table 1:** Alignment of structural proteins in SARS-CoV-1 and SARS-CoV-2.

SARS-CoV-1	SARS-CoV-2	BPO	CCO	MFO
O00303	P48556	0.289	0.860	
Q9BYF1	Q7L8L6	0.363	0.749	0.165
O95295	Q7Z5G4	0.485	0.855	
(a) Spike protein S (P59594, P0DTC2)				
SARS-CoV-1	SARS-CoV-2	BPO	CCO	MFO
Q07817	Q9Y3A6	0.299	0.806	
O00560	Q6UX04	0.152	0.735	0.104
(b) Envelope protein E (P59637, P0DTC4)				
SARS-CoV-1	SARS-CoV-2	BPO	CCO	MFO
O14920	Q9UHD2	0.863	0.839	1.000
P00403	50Q96HR9	0.077	0.816	
Q8TEB7	50Q96ER3		0.652	
O00303	50P48556	0.289	0.860	
P69849	50Q9NQC3		0.873	
Q9BYF1	50Q7L8L6	0.363	0.749	0.165
Q03135	50Q00765		0.801	
(c) Membrane protein M (P59596, P0DTC5)				
SARS-CoV-1	SARS-CoV-2	BPO	CCO	MFO
P68104	Q9HD40	0.625	0.748	0.511
B8ZZN6	Q9Y3U8			
Q92499	Q9NR30	0.558	0.941	0.854
Q53GL0	Q8TAD8	0.131	0.851	
P09651	Q8NCA5	0.090	0.692	0.631
P14618	Q13310	0.634	0.814	0.565
P62937	P67870	0.679	0.916	0.262
B0QYN7	P19784			
Q9Y4W2	Q9NW13	0.622	0.917	0.674
Q9HCD5	P11940	0.055	0.869	0.868
(d) Nucleocapsid protein N (P59595, P0DTC9)				

**Table 2:** Alignment of non-structural proteins in SARS-CoV-1 and SARS-CoV-2.

SARS-CoV-1	SARS-CoV-2	BPO	CCO	MFO
O43447	Q9H2H8	0.723	0.929	0.694
P62937	P67870	0.679	0.916	0.262
P62942	Q15370	0.470	0.863	0.083
Q13427	Q4V328	0.064	0.862	
Q9UKA8	Q9Y680	0.049	0.848	0.209
Q9Y4W2	Q9NW13	0.622	0.917	0.674
P28340	P09884	0.887	0.965	0.792
(a) Non-structural protein nsp1 (NP_828860, P0DTD1-PRO_0000449619)				
SARS-CoV-1	SARS-CoV-2	BPO	CCO	MFO
P05155	Q9GZU3			
Q8WXF8	Q99988	0.367	0.735	
Q7Z3Q1	Q6NXT4	1.000	0.436	
Q7Z494	Q08378	0.385	0.756	
P13796	P52306	0.439	0.852	0.116
P02768	O14975	0.457	0.869	0.440
(b) Non-structural protein nsp2 (NP_828861, P0DTD1-PRO_0000449620)				
SARS-CoV-1	SARS-CoV-2	BPO	CCO	MFO
P08949	Q96DA6	0.372	0.539	
Q9Y4W2	Q9NW13	0.622	0.917	0.674
O95865	Q9BSF4	0.092	0.772	
(c) Non-structural protein nsp4 (NP_904322, P0DTD1-PRO_0000449622)				
SARS-CoV-1	SARS-CoV-2	BPO	CCO	MFO
P23025	Q8NEJ9	0.353	0.887	0.419
P62942	Q15370	0.470	0.863	0.083
O75348	O75347	0.104	0.713	0.201
Q92802	O95391		0.811	0.209
Q9GZN8	P82663			
(d) Non-structural protein nsp5 (NP_828863, P0DTD1-PRO_0000449623)				
SARS-CoV-1	SARS-CoV-2	BPO	CCO	MFO
O14498	Q9BQQ3	0.407	0.792	
(e) Non-structural protein nsp6 (NP_828864, P0DTD1-PRO_0000449624)				
SARS-CoV-1	SARS-CoV-2	BPO	CCO	MFO
A9UHW6	Q8WVC6			0.141
O95865	Q9BSF4	0.092	0.772	
P13796	P52306	0.439	0.852	0.116
P49703	P62330	0.464	0.749	0.852
Q13564	Q96K12	0.172	0.741	0.286
Q53GL0	Q8TAD8	0.131	0.851	
Q8TEB7	Q96ER3		0.652	
Q92560	Q96CN9		0.808	
P62258	O00124	0.249	0.722	
Q9HCD5	P11940	0.055	0.869	0.868
A9UHW6	Q8WVC6			0.141
P08949	Q96DA6	0.372	0.539	
P50583	Q96A26	0.826	0.968	
P20290	P51148	0.521	0.666	0.138
P62879	P62873	0.455	0.981	0.879
Q99426	P62820	0.289	0.761	
Q16548	P61006	0.381	0.702	
Q92843	P11233	0.485	0.727	
Q56VL3	O43169		0.869	
(f) Non-structural protein nsp7 (NP_828865, P0DTD1-PRO_0000449625)				
SARS-CoV-1	SARS-CoV-2	BPO	CCO	MFO
P05155	Q9GZU3			
P54274	Q9H173	0.200	0.655	0.216
P69849	Q9NQC3		0.873	
P23588	O00566	0.477	0.701	0.917
Q9P0M6	O14745	0.535	0.804	0.102
P68104	Q9HD40	0.625	0.748	0.511
P49069	Q13868	0.088	0.762	0.080
Q96GS6	Q9Y399	0.361	0.766	
P10415	Q9NQT5	0.323	0.887	0.234
P23025	Q8NEJ9	0.353	0.887	0.419
Q9Y2D1	Q9H6F5	0.108	0.772	0.271
Q9GZN8	P82663			
Q13064	O95260	0.714	0.234	0.083
P23588	O00566	0.477	0.701	0.917
(g) Non-structural protein nsp8 (NP_828866, P0DTD1-PRO_0000449626)				

**Table 3:** Alignment of non-structural proteins in SARS-CoV-1 and SARS-CoV-2.

SARS-CoV-1	SARS-CoV-2	BPO	CCO	MFO
P06733	P31323	0.310	0.906	0.539
Q5SQN1	Q86VR2	0.112	0.371	
Q6P587	P13984	0.255	0.828	0.457
Q7Z494	Q08378	0.385	0.756	
Q9BUV0	P09601			
Q9UQN3	O43633	0.932	0.885	
P25685	Q9NZL9	0.179	0.912	0.879
P09630	Q15056	0.117	0.791	0.387
Q9UMX0	Q9BVL2	0.329	0.779	0.170
Q6P587	P13984	0.255	0.828	0.457
(a) Non-structural protein nsp9 (NP_828867, P0DTD1-PRO_0000449627)				
SARS-CoV-1	SARS-CoV-2	BPO	CCO	MFO
P09630	Q15056	0.117	0.791	0.387
P30876	Q66GS9	0.137	0.679	
P51948	Q9H4P4	0.378	0.835	0.420
Q5SQN1	Q86VR2	0.112	0.371	
Q8TD31	Q9UJC3	0.729	0.924	
O95299	O14656	0.378	0.699	0.129
Q99426	P62820	0.289	0.761	
Q99471	Q8N6S5			
Q9UQN3	O43633	0.932	0.885	
O43447	Q9H2H8	0.723	0.929	0.694
P46379	Q5T6F2		0.799	0.102
Q9H000	Q96IZ5	0.188		0.733
Q92802	O95391		0.811	0.209
P98161	O75592	0.379	0.725	0.596
(b) Non-structural protein nsp12 (NP_828869, P0DTD1-PRO_0000449629)				
SARS-CoV-1	SARS-CoV-2	BPO	CCO	MFO
P20290	P51148	0.521	0.666	0.138
P35354	Q96AY3	0.100	0.895	0.607
Q9Y2D1	Q9H6F5	0.108	0.772	0.271
Q8N488	Q9HAV7	0.202	0.932	0.190
(c) Non-structural protein nsp10 (NP_828868, P0DTD1-PRO_0000449628)				
SARS-CoV-1	SARS-CoV-2	BPO	CCO	MFO
P62937	P67870	0.679	0.916	0.262
P51948	Q9H4P4	0.378	0.835	0.420
P49703	P62330	0.464	0.749	0.852
(d) Non-structural protein nsp15 (NP_828872, P0DTD1-PRO_0000449632)				

**Table 4:** Alignment of accessory proteins in SARS-CoV-1 and SARS-CoV-2.

SARS-CoV-1	SARS-CoV-2	BPO	CCO	MFO
Q13561	Q8TD10		0.652	
P62258	O00124	0.249	0.722	
Q03135	Q00765		0.801	
Q99471	Q8N6S5			
Q9BUV0	P09601			
(a) Accessory protein orf3a (P59632, P0DTC3)				
SARS-CoV-1	SARS-CoV-2	BPO	CCO	MFO
P25685	Q9NZL9	0.179	0.912	0.879
Q13561	Q8TD10		0.652	
Q5TBA9	P49454	0.383	0.806	0.100
P52292	Q9NZJ7	0.152	0.808	
Q13287	P78406	0.174	0.825	
Q13009	P52948	0.169	0.829	0.170
(b) Accessory protein orf6 (P59634, P0DTC6)				
SARS-CoV-1	SARS-CoV-2	BPO	CCO	MFO
P10415	Q9NQT5	0.323	0.887	0.234
P50583	Q96A26	0.826	0.968	
Q07817	Q9Y3A6	0.299	0.806	
Q16548	P61006	0.381	0.702	
Q92843	P11233	0.485	0.727	
O43765	Q7Z4Q2	0.489	0.531	
(c) Accessory protein orf7a (P59635, P0DTC7)				
SARS-CoV-1	SARS-CoV-2	BPO	CCO	MFO
P08708	Q9H773	0.509	0.861	0.185
P25787	Q9H2P9	0.319	0.701	0.120
Q9UQN3	O43633	0.932	0.885	
Q9P0M6	O14745	0.535	0.804	0.102
(d) Accessory protein orf9b (P59636, P0DTD2)				

**Table 5:** Human proteins that interact with SARS-CoV-1 and SARS-CoV-2 structural proteins, whose alignment would preserve virus-host interactions.

SARS-CoV-1	SARS-CoV-2	BPO	CCO	MFO
Q9BYF1	Q7Z5G4	0.520	0.859	0.137
O00303	Q7Z5G4	0.373	0.747	0.098
O00303	Q9C0B5	0.487	0.601	0.098
Q9BYF1	Q9C0B5	0.360	0.650	0.137
(a) Spike protein S (P59594, P0DTC2)				
SARS-CoV-1	SARS-CoV-2	BPO	CCO	MFO
Q07817	O00203	0.548	0.805	1.000
Q07817	O60885	0.539	0.770	0.566
O00560	O00203	0.666	0.801	0.335
Q07817	P25440	0.402	0.759	0.397
O00560	Q8IWA5	0.531	0.864	0.105
O00560	P25440	0.353	0.734	0.335
(b) Envelope protein E (P59637, P0DTC4)				
SARS-CoV-1	SARS-CoV-2	BPO	CCO	MFO
O14920	Q96D53	0.310	0.889	0.852
O14920	Q9ULX6	0.246	0.880	0.638
O14920	P05026	0.472	0.743	0.549
O14920	P27105	0.473	0.712	0.574
O14920	Q7L8L6	0.308	0.865	0.449
O14920	P38606	0.284	0.842	0.491
(c) Membrane protein M (P59596, P0DTC5)				
SARS-CoV-1	SARS-CoV-2	BPO	CCO	MFO
P09651	Q8TAD8	0.630	0.891	0.906
P09651	P11940	0.436	0.954	0.906
P09651	Q6PKG0	0.572	0.786	0.906
P14618	P67870	0.693	0.909	0.657
P09651	Q9UN86	0.616	0.802	0.774
P09651	Q13310	0.425	0.855	0.906
(d) Nucleocapsid protein N (P59595, P0DTC9)				

We can observe that the four structural proteins in one network were aligned with the corresponding protein in the other network. Also, most of the non-structural proteins and half of the accessory proteins in one network were aligned with the corresponding protein in the other network. On the other hand, for each pair of aligned viral proteins, the highlighted proteins in the same column of a viral protein are the human proteins it interacts with. For instance, human proteins O00303 and Q9BYF1 interact with the SARS-CoV-1 spike protein P59594, while human protein Q7Z5G4 interacts with the SARS-CoV-2 spike protein P0DTC2. Table 1(a) shows that O00303 is aligned with P48556, Q9BYF1 is aligned with Q7L8L6, and O95295 is aligned with Q7Z5G4. Missing data are due to lack of GO term annotation for the two interacting proteins.

As can be seen in these tables, most of the aligned proteins have a cellular component ontology score above 0.700. This means that, despite the low number of conserved interactions, the aligned proteins share their cellular location. For instance, those human proteins that interact with the spike protein in SARS-CoV-1 are aligned with human proteins that interact with the membrane protein in SARS-CoV-2. However, some biological process ontology scores between aligned human proteins are very low. This can be explained by the lack of biological process ontology GO term annotation for one of the two interacting proteins.

With respect to molecular function ontology, it is remarkable that we obtained high scores for aligning proteins that interact with structurally different viral proteins. Indeed, one of the measures used to test the correctness of a protein-protein interaction network alignment is the *edge correctness* score, which measures the ratio of conserved edges in a given alignment. Edge correctness assume that one of the aims of the alignment is to find similar regions between the two aligned networks, in terms of network topology. In the context of protein-protein interaction networks, it is also assumed that two proteins interact when they together carry out some biological function. For virus-host protein-protein interaction networks, viral proteins interact with host proteins to perturb the intracellular networks of their hosts to their advantage, and many virus-host interactions occur at the level of physical protein-protein interactions [7]. This means that a viral protein interacts with a host protein to carry out a cellular process, and this pathway of virus-host interactions constitutes the infection mechanism of the virus.

The question then arises, when a viral protein in one network is aligned with a viral protein in another network, should the host proteins that interact with one viral protein be aligned with those host proteins that interact with the other viral protein? Clearly the answer is yes, when the viral-host protein-protein interaction is a similar infectious process stage. Therefore, aligned virus-host interactions must entail conserved stages in the infectious process. However, non-conserved edges do not necessarily imply incorrect alignments. Indeed, when we analyze in more depth the functional description [3] of the aligned human proteins that interact with Coronavirus proteins, we realize that they share a function related to viral infection, although their alignment introduces a non-preserved interaction. This is the case of the following pairs of proteins:

### O14920 and Q9UHD2

The molecular function ontology score of these proteins is 1.000. Human protein O14920 interacts with viral protein P59596 (membrane) of SARS-CoV-1, which is aligned with protein P0DTC5 (membrane) of SARS-CoV-2. On the other hand, human protein Q9UHD2 interacts with viral protein P0DTD1-PRO_0000449630 (orf9b) of SARS-CoV-2. However, the functional description of the aligned human proteins reflects correctness of the consensus alignment:

- O14920: Serine kinase that plays an essential role in the NF-kappa-B signaling pathway which is activated by multiple stimuli such as inflammatory cytokines, bacterial or viral products.
- Q9UHD2: Serine/threonine kinase that plays an essential role in regulating inflammatory responses to foreign agents. Following activation of toll-like receptors by viral or bacterial components, associates with TRAF3 and TANK and phosphorylates interferon regulatory factors (IRFs) IRF3 and IRF7 as well as DDX3X.

### Q92499 and Q9NR30

The molecular function ontology score of these proteins is 0.850. Human protein Q9NR30 interacts with viral protein P0DTC9 (nucleocapsid) of SARS-CoV-2, which is aligned with protein P59595 (nucleocapsid) of SARS-CoV-1. On the other hand, human protein Q92499 interacts with viral protein P0C6X7-PRO_0000037320 (proofreading exoribonuclease in replicase polyprotein 1ab) of SARS-CoV-1. The functional description of the aligned human proteins is:

- Q92499: Helicase required for Coronavirus IBV replication. Antiviral defense.
- Q9NR30: Component of a multi-helicase-TICAM1 complex that acts as a cytoplasmic sensor of viral double-stranded RNA (dsRNA) and plays a role in the activation of a cascade of antiviral responses including the induction of proinflammatory cytokines via the adapter molecule TICAM1.

### P49703 and P62330

The molecular function ontology score of these proteins is 0.850. Both are GTP-binding proteins. Human protein P49703 interacts with viral protein NP_828865 (nsp7) of SARS-CoV-1. Human protein P62330 interacts with viral protein P0DTD1-PRO0000449632 (nsp15) of SARS-CoV-2, which, as reported in [4], “has uridine-specific endoribonuclease (endoU) activity and is essential for viral RNA synthesis,” with the endoU domain being “one of the most conserved proteins among CoVs and related viruses, suggesting important functions in the viral replicative cycle.” The functional description of the aligned human proteins is:

- P49703: Small GTP-binding protein which cycles between an inactive GDP-bound and an active GTP-bound form, and the rate of cycling is regulated by guanine nucleotide exchange factors (GEF) and GTPase-activating proteins (GAP).
- P62330: GTP-binding protein involved in protein trafficking that regulates endocytic recycling and cytoskeleton remodeling. Activation is generally mediated by a guanine exchange factor (GEF), while inactivation through hydrolysis of bound GTP is catalyzed by a GTPase activating protein (GAP).

Therefore, it is not clear whether edge preservation should always be required in a correct alignment of virus-host protein-protein interaction networks. To reinforce this idea, we considered the functional similarity of all pairs of human proteins whose alignment would preserve edges, given the consensus alignment of 24 viral proteins. For each pair of aligned viral proteins (say, membrane proteins) we considered the biological process, the cell component, and the molecular function ontology scores of all pairs of human proteins that interact with the aligned viral proteins (say, all pairs of human proteins such that the first protein interacts with viral membrane protein P59596 of SARS-CoV-1 and the second protein interacts with viral membrane protein P0DTC5 of SARS-CoV-2). The cellular component ontology score is above 0.800 for most of the aligned human proteins, but the highest molecular function ontology score is 0.852, while it is 1.000 in the consensus alignment, and the highest biological process ontology score of the aligned human proteins is 0.670, while it is 0.863 in the consensus alignment.

Nevertheless, some of these pairs of human proteins whose alignment would preserve virus-host interactions do show high functional similarity scores, and it could be interesting to further study their role in the viral mechanism of host infection. Table 5 shows some of the highest ranking pairs of human proteins across biological process, cellular component, and molecular function ontology scores for the structural viral proteins in the consensus alignment, in descending order of average score. See the Supplementary material for more details.

We observed that, based on current knowledge, SARS-CoV-1 and SARS-CoV-2 share only 6 human proteins in their virus-host protein-protein interaction networks. On the one hand, aligned viral proteins in the consensus alignment obtained with our method show a sequence similarity of over 75% on the average, and most of the SARS-CoV-1 proteins are aligned with SARS-CoV-2 proteins that belong to the same category (spike, envelope, membrane, nucleocapsid, and the various non-structural and accessory proteins) in the genome organization of the viruses. On the other hand, the proposed alignment method does not preserve the virus-host interactions. This suggests that these viruses, despite their classification as human pathogens within the *Coronaviridae* family, do not follow the same detailed mechanism of host infection. We believe that further research on these aligned human proteins with high molecular function ontology scores, will help to elucidate the viral mechanism of infection and replication that is necessary to accomplish the goal of antiviral drug or vaccine discovery.

## Supporting information

S1 File (PDF)

S2 File (GZ)

S3 File (GZ)

S4 File (GZ)

S5 File (GZ)

S6 File (MOD)
