# Supplementary material for: Alignment of virus-host protein-protein interaction networks by integer linear programming: SARS-CoV-2
Source: PLoS One. 2020 Dec 7;15(12):e0236304. doi: 10.1371/journal.pone.0236304 (PMC7721128; doi:10.1371/journal.pone.0236304)
Supplement: S1 File — (PDF) [file pone.0236304.s001.pdf]

Supplementary Material to “Alignment of Virus-Host Protein-Protein Interaction Networks by Integer Linear Programming: SARS-CoV-2”

Mercè Llabrés
Department of Mathematics and Computer Science
University of the Balearic Islands

Gabriel Valiente
Algorithms, Bioinformatics, Complexity and Formal Methods
Research Group, Technical University of Catalonia

Human proteins that interact with SARS-CoV-1 and SARS-CoV-2 structural proteins, whose alignment would preserve virus-host interactions. For each pair of viral protein in the consensus alignment of the virus-host protein-protein interaction networks for SARS-CoV-1 and SARS-CoV-2, we show the molecular function ontology (MFO) score, the biological process ontology (BPO) score, and the cellular component ontology (CCO) score of the human proteins they interact with, in decreasing order of average score. Missing data is due to lack of GO term annotation for the two interacting proteins.

A Structural Proteins

Spike

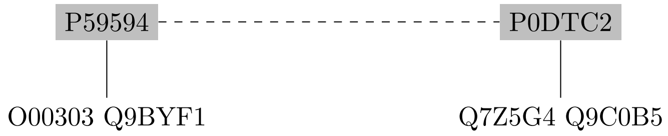

SARS-CoV-1	SARS-CoV-2	BPO	CCO	MFO
Q9BYF1	Q7Z5G4	0.520	0.859	0.137
O00303	Q7Z5G4	0.373	0.747	0.098
O00303	Q9C0B5	0.487	0.601	0.098
Q9BYF1	Q9C0B5	0.360	0.650	0.137

Envelope

P59637 - - - - P0DTC4

P59637: O00560 Q07817

P0DTC4: O00203 O60885 P25440 Q6UX04 Q86VM9 Q8IWA5

SARS-CoV-1	SARS-CoV-2	BPO	CCO	MFO
Q07817	O00203	0.548	0.805	1.000
Q07817	O60885	0.539	0.770	0.566
O00560	O00203	0.666	0.801	0.335
Q07817	P25440	0.402	0.759	0.397
O00560	Q8IWA5	0.531	0.864	0.105
O00560	P25440	0.353	0.734	0.335
O00560	O60885	0.226	0.751	0.317
O00560	Q86VM9		0.735	0.515
Q07817	Q8IWA5	0.367	0.699	0.085
Q07817	Q6UX04	0.164	0.762	0.083
O00560	Q6UX04	0.152	0.735	0.104
Q07817	Q86VM9		0.762	0.209

Membrane

P59596 - - - - P0DTC5

P59596: O14920

P0DTC5: O75439 O95070 P05026 P11310 P13804 P27105 P38435 P38606 P48556 Q00765 Q10713 Q4KMQ2 Q5JRX3 Q6PML9 Q7L8L6 Q8NEW0 Q96CW5 Q96D53 Q96ER3 Q96HR9 Q96HW7 Q9BQT8 Q9BSJ2 Q9BW92 Q9NQC3 Q9UBU6 Q9UDR5 Q9ULX6 Q9Y312 Q9Y6E2

SARS-CoV-1	SARS-CoV-2	BPO	CCO	MFO
O14920	Q96D53	0.310	0.889	0.852
O14920	Q9ULX6	0.246	0.880	0.638
O14920	P05026	0.472	0.743	0.549
O14920	P27105	0.473	0.712	0.574
O14920	Q7L8L6	0.308	0.865	0.449

SARS-CoV-1	SARS-CoV-2	BPO	CCO	MFO
O14920	P38606	0.284	0.842	0.491
O14920	Q9NQC3	0.236	0.873	0.499
O14920	P48556	0.670	0.935	
O14920	O75439	0.210	0.865	0.499
O14920	P11310	0.172	0.829	0.541
O14920	Q9BW92	0.192	0.801	0.541
O14920	P38435	0.568	0.798	0.161
O14920	Q9BSJ2	0.363	0.881	0.273
O14920	P13804	0.170	0.801	0.541
O14920	Q96CW5	0.363	0.863	0.283
O14920	Q10713	0.210	0.788	0.499
O14920	Q5JRX3	0.215	0.801	0.438
O14920	Q4KMQ2	0.444	0.861	0.099
O14920	Q6PML9	0.321	0.805	0.237
O14920	O95070	0.441	0.759	
O14920	Q9UDR5	0.206	0.801	0.161
O14920	Q9Y312	0.188	0.889	
O14920	Q9BQT8	0.157	0.801	0.099
O14920	Q96HW7	0.150	0.863	
O14920	Q8NEW0	0.109	0.778	0.099
O14920	Q96HR9	0.089	0.827	
O14920	Q9Y6E2	0.137	0.743	
O14920	Q00765		0.798	
O14920	Q96ER3		0.741	

Nucleocapsid

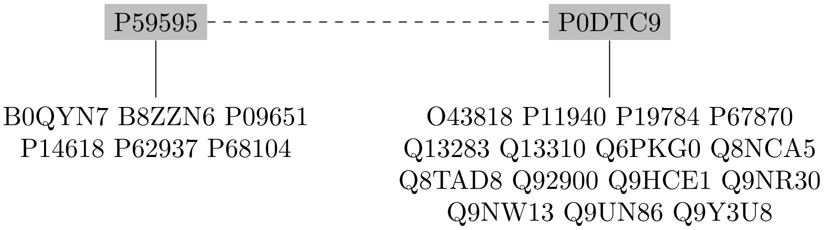

SARS-CoV-1	SARS-CoV-2	BPO	CCO	MFO
P09651	Q8TAD8	0.630	0.891	0.906
P09651	P11940	0.436	0.954	0.906
P09651	Q6PKG0	0.572	0.786	0.906
P14618	P67870	0.693	0.909	0.657
P09651	Q9UN86	0.616	0.802	0.774
P09651	Q13310	0.425	0.855	0.906
P14618	P19784	0.505	0.800	0.840

SARS-CoV-1	SARS-CoV-2	BPO	CCO	MFO
P09651	Q92900	0.658	0.855	0.603
P68104	Q9NR30	0.596	0.777	0.726
P09651	Q9NW13	0.477	0.825	0.783
P09651	Q13283	0.583	0.855	0.625
P68104	Q92900	0.548	0.784	0.703
P68104	P11940	0.634	0.871	0.524
P14618	Q13310	0.634	0.814	0.565
P68104	Q9Y3U8	0.723	0.807	0.449
P62937	Q9UN86	0.560	0.844	0.565
P09651	Q9Y3U8	0.584	0.823	0.556
P68104	Q13310	0.644	0.791	0.524
P09651	O43818	0.314	0.859	0.783
P14618	Q9NR30	0.555	0.777	0.616
P14618	Q92900	0.583	0.773	0.588
P62937	P11940	0.412	0.975	0.554
P14618	P11940	0.444	0.917	0.565
P14618	Q13283	0.570	0.814	0.539
P68104	Q9HCE1	0.323	0.871	0.723
P09651	Q9HCE1	0.475	0.954	0.479
P62937	Q13310	0.461	0.884	0.554
P14618	Q9UN86	0.589	0.776	0.523
P14618	Q9HCE1	0.368	0.917	0.597
P62937	Q13283	0.550	0.884	0.446
P62937	Q92900	0.597	0.826	0.449
P62937	Q9Y3U8	0.578	0.839	0.452
P62937	P67870	0.679	0.916	0.262
P68104	Q13283	0.393	0.791	0.646
P62937	Q8TAD8	0.404	0.864	0.554
P68104	P67870	0.538	0.894	0.384
P62937	Q9HCE1	0.395	0.975	0.438
P68104	Q6PKG0	0.574	0.706	0.524
P62937	Q6PKG0	0.463	0.786	0.554
P09651	Q9NR30	0.444	0.811	0.513
P14618	Q9Y3U8	0.523	0.782	0.448
P14618	Q6PKG0	0.447	0.727	0.565
P62937	Q9NR30	0.464	0.782	0.477
P68104	Q9UN86	0.429	0.748	0.543
P68104	Q8TAD8	0.373	0.790	0.524
P68104	Q9NW13	0.407	0.757	0.511
P14618	Q8TAD8	0.283	0.801	0.565
P68104	O43818	0.371	0.751	0.511
P62937	Q9NW13	0.299	0.783	0.542
P62937	P19784	0.514	0.854	0.234
P62937	O43818	0.276	0.768	0.542
P68104	P19784	0.318	0.841	0.396

SARS-CoV-1	SARS-CoV-2	BPO	CCO	MFO
P14618	Q9NW13	0.271	0.726	0.556
P14618	O43818	0.277	0.716	0.556
P09651	P67870	0.408	0.897	0.136
P62937	Q8NCA5	0.228	0.679	0.508
P09651	Q8NCA5	0.090	0.692	0.631
P09651	P19784	0.310	0.829	0.154
P14618	Q8NCA5	0.111	0.618	0.524
P68104	Q8NCA5	0.166	0.607	0.453

B Non-structural Proteins

Nsp1

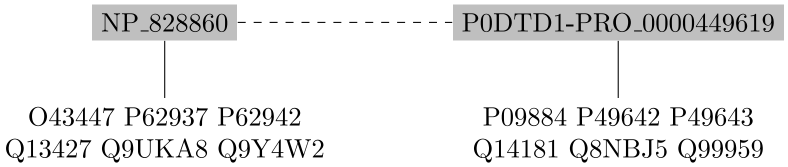

SARS-CoV-1	SARS-CoV-2	BPO	CCO	MFO
O43447	Q99959	0.447	0.931	0.292
P62937	P09884	0.503	0.797	0.365
Q13427	Q14181	0.408	0.906	0.320
P62937	Q14181	0.462	0.826	0.320
Q9Y4W2	Q14181	0.361	0.941	0.302
Q9UKA8	Q99959	0.382	0.699	0.520
Q13427	P49642	0.408	0.835	0.337
Q13427	P09884	0.339	0.871	0.365
P62937	P49642	0.462	0.751	0.337
O43447	P49643	0.376	0.914	0.242
Q9Y4W2	P49642	0.361	0.883	0.282
O43447	P49642	0.376	0.914	0.214
O43447	P09884	0.332	0.889	0.267
Q9Y4W2	P09884	0.287	0.938	0.252
Q13427	P49643	0.408	0.835	0.234
O43447	Q14181	0.376	0.926	0.162
P62937	P49643	0.462	0.751	0.234
Q9Y4W2	P49643	0.361	0.883	0.138
P62942	Q99959	0.445	0.853	0.083
P62937	Q99959	0.257	0.795	0.245
Q13427	Q99959	0.183	0.866	0.245
Q9UKA8	P09884	0.143	0.641	0.492

SARS-CoV-1	SARS-CoV-2	BPO	CCO	MFO
P62942	Q14181	0.222	0.811	0.169
P62942	Q8NBJ5		0.876	0.272
P62942	P49643	0.222	0.733	0.187
P62942	P09884	0.218	0.768	0.142
Q9Y4W2	Q99959	0.146	0.899	0.080
P62942	P49642	0.222	0.733	0.156
Q9UKA8	Q14181	0.143	0.686	0.239
Q9UKA8	P49642	0.143	0.627	0.295
O43447	Q8NBJ5		0.807	0.198
Q13427	Q8NBJ5		0.836	0.157
Q9UKA8	P49643	0.143	0.627	0.201
Q9UKA8	Q8NBJ5		0.848	0.067
P62937	Q8NBJ5		0.731	0.157
Q9Y4W2	Q8NBJ5		0.762	0.090

Nsp2

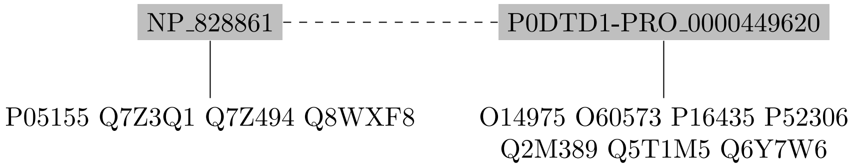

SARS-CoV-1	SARS-CoV-2	BPO	CCO	MFO
P05155	P52306	0.276	0.867	1.000
P05155	O14975	0.545	0.874	0.128
Q8WXF8	Q6Y7W6	0.440	0.673	0.392
Q8WXF8	P16435	0.487	0.748	0.248
Q8WXF8	O60573	0.371	0.655	0.433
P05155	Q5T1M5	0.496	0.776	0.123
Q7Z494	P52306	0.478	0.881	
Q7Z494	Q6Y7W6	0.540	0.773	
P05155	Q2M389	0.542	0.722	
Q7Z494	O60573	0.402	0.781	
Q7Z3Q1	Q2M389	0.795	0.387	
P05155	Q6Y7W6	0.363	0.727	0.080
Q7Z494	O14975	0.331	0.834	
Q8WXF8	O14975	0.288	0.638	0.220
Q7Z494	Q2M389	0.360	0.768	
Q7Z3Q1	Q5T1M5	0.701	0.425	
Q7Z494	Q5T1M5	0.318	0.785	
Q8WXF8	Q5T1M5	0.110	0.736	0.223
Q8WXF8	P52306	0.270	0.678	0.088

SARS-CoV-1	SARS-CoV-2	BPO	CCO	MFO
Q7Z3Q1	O14975	0.443	0.566	
P05155	P16435	0.134	0.722	0.125
Q7Z494	P16435	0.116	0.846	
P05155	O60573	0.176	0.652	0.089
Q8WXF8	Q2M389	0.060	0.769	
Q7Z3Q1	P52306	0.056	0.624	
Q7Z3Q1	Q6Y7W6	0.088	0.378	
Q7Z3Q1	P16435	0.061	0.399	
Q7Z3Q1	O60573	0.066	0.239	

Nsp4

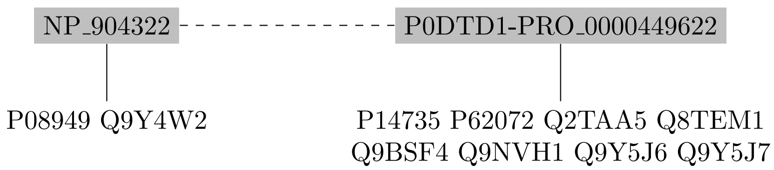

SARS-CoV-1	SARS-CoV-2	BPO	CCO	MFO
Q9Y4W2	P14735	0.244	0.806	0.223
Q9Y4W2	Q9Y5J7	0.101	0.827	0.156
Q9Y4W2	P62072	0.101	0.827	0.156
P08949	P14735	0.490	0.583	
Q9Y4W2	Q9Y5J6	0.079	0.827	0.156
Q9Y4W2	Q2TAA5	0.175	0.762	0.090
Q9Y4W2	Q9NVH1	0.161	0.827	
Q9Y4W2	Q8TEM1	0.085	0.864	
Q9Y4W2	Q9BSF4	0.107	0.827	
P08949	Q9BSF4	0.382	0.539	
P08949	Q9Y5J6	0.378	0.539	
P08949	Q9Y5J7	0.351	0.539	
P08949	P62072	0.351	0.539	
P08949	Q8TEM1	0.366	0.498	
P08949	Q2TAA5	0.093	0.568	
P08949	Q9NVH1	0.094	0.539	

Nsp5

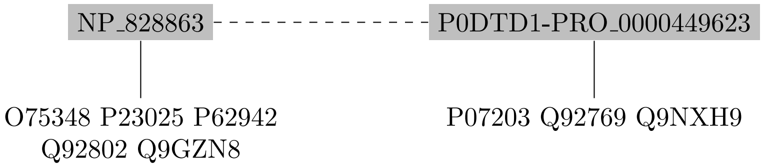

SARS-CoV-1	SARS-CoV-2	BPO	CCO	MFO
P23025	Q92769	0.432	0.964	0.485
P23025	Q9NXH9	0.359	0.822	0.625
P62942	P07203	0.362	0.857	0.387
P62942	Q92769	0.499	0.779	0.139
Q92802	Q92769		0.788	0.442
P23025	P07203	0.402	0.717	0.101
O75348	Q92769	0.101	0.713	0.388
O75348	P07203	0.183	0.767	0.141
P62942	Q9NXH9	0.133	0.711	0.155
Q92802	Q9NXH9		0.778	0.166
O75348	Q9NXH9	0.058	0.639	0.145
Q92802	P07203		0.690	0.083

Nsp6

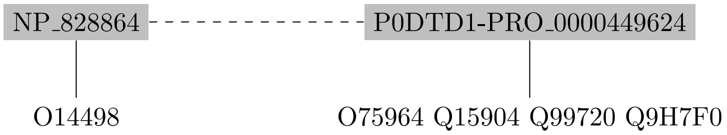

SARS-CoV-1	SARS-CoV-2	BPO	CCO	MFO
O14498	Q15904	0.530	0.872	
O14498	O75964	0.381	0.756	
O14498	Q9H7F0	0.588	0.518	
O14498	Q99720	0.346	0.749	

Nsp7

NP_828865 - - - - - - - - - - P0DTD1-PRO_0000449625

A9UHW6 O95865 P13796
P49703 P62258 Q13564 Q53GL0
Q8TEB7 Q92560 Q9HCD5

O00116 O43169 O95573 P00387
P11233 P21964 P51148 P51149
P61006 P61019 P61026 P61106 P61586
P62820 P62873 P63218 Q12907
Q13724 Q5JTV8 Q5VT66 Q6ZRP7
Q7LGA3 Q8N183 Q8NBX0 Q8WTV0
Q8WUY8 Q8WVC6 Q96A26
Q96DA6 Q9BQE4 Q9H7Z7 Q9NP72

SARS-CoV-1	SARS-CoV-2	BPO	CCO	MFO
P49703	Q9NP72	0.700	0.811	1.000
P49703	P51148	0.776	0.731	1.000
P49703	P61019	0.759	0.728	1.000
P49703	Q12907	1.000	0.755	0.685
P13796	P11233	0.620	0.886	0.865
P13796	P61586	0.620	0.881	0.865
P13796	P62873	0.589	0.909	0.719
P62258	Q9BQE4	0.604	0.910	0.701
P13796	P61006	0.457	0.872	0.865
P62258	P61586	0.762	0.926	0.480
P49703	P51149	0.458	0.697	1.000
P13796	P61106	0.565	0.811	0.742
P49703	P61106	0.542	0.706	0.852
P13796	P61026	0.472	0.875	0.742
P62258	P61006	0.690	0.879	0.480
P62258	P11233	0.676	0.877	0.480
P49703	P61026	0.473	0.706	0.852
P49703	P62820	0.328	0.689	1.000
P49703	O00116	0.621	0.878	0.489
P13796	Q9BQE4	0.364	0.956	0.665
O95865	Q8WTV0	0.521	0.814	0.615
O95865	P00387	0.478	0.813	0.636
P49703	P11233	0.368	0.789	0.754
P62258	P62873	0.530	0.874	0.504
P49703	P61006	0.437	0.715	0.754
P13796	P62820	0.570	0.818	0.516
Q13564	Q9BQE4	0.433	0.776	0.694
Q9HCD5	Q6ZRP7	1.000	0.837	0.064
Q8TEB7	Q12907	0.067	0.834	1.000
O95865	Q5VT66	0.457	0.803	0.636
P13796	Q8WTV0	0.466	0.846	0.581
Q8TEB7	P61019	0.346	0.847	0.685

SARS-CoV-1	SARS-CoV-2	BPO	CCO	MFO
O95865	O95573	0.545	0.764	0.561
P13796	P51149	0.487	0.864	0.516
O95865	P21964	0.419	0.879	0.560
Q8TEB7	P21964	0.418	0.743	0.689
Q8TEB7	P62820	0.252	0.904	0.685
P13796	O95573	0.387	0.681	0.753
Q8TEB7	Q8WTV0	0.261	0.766	0.793
O95865	O00116	0.489	0.783	0.541
Q8TEB7	O43169		0.804	1.000
P62258	P62820	0.779	0.838	0.187
P13796	Q9NP72	0.507	0.777	0.516
O95865	Q9H7Z7	0.483	0.829	0.469
O95865	P61106	0.446	0.799	0.536
P62258	P51149	0.710	0.878	0.187
Q8TEB7	P00387	0.237	0.831	0.706
P13796	P21964	0.311	0.932	0.518
O95865	P62820	0.305	0.849	0.605
Q13564	P62873	0.416	0.791	0.551
P49703	P61586	0.220	0.781	0.754
P13796	P51148	0.415	0.821	0.516
O95865	P51149	0.353	0.790	0.605
P62258	Q8WTV0	0.637	0.858	0.247
O95865	P61006	0.362	0.887	0.491
Q8TEB7	P61006	0.346	0.866	0.516
P49703	Q8WTV0	0.364	0.756	0.608
O95865	P61019	0.294	0.825	0.605
Q8TEB7	P51149	0.224	0.813	0.685
Q8TEB7	Q9NP72	0.113	0.922	0.685
P62258	P61026	0.574	0.887	0.256
Q8TEB7	Q5VT66	0.202	0.804	0.706
O95865	P61586	0.350	0.862	0.491
O95865	P51148	0.295	0.794	0.605
P49703	P00387	0.419	0.680	0.590
Q13564	O95573	0.151	0.672	0.852
P62258	P61106	0.579	0.837	0.256
Q8TEB7	O95573	0.226	0.847	0.595
P49703	Q8NBX0	0.662	0.842	0.160
P62258	O95573	0.427	0.722	0.509
Q13564	P11233	0.408	0.735	0.503
O95865	Q9NP72	0.281	0.749	0.605
Q13564	P61586	0.414	0.713	0.503
P13796	Q5JTV8	0.249	0.646	0.733
P49703	Q9H7Z7	0.452	0.757	0.417
O95865	Q13724	0.435	0.871	0.307
Q8TEB7	P61106	0.182	0.848	0.579

SARS-CoV-1	SARS-CoV-2	BPO	CCO	MFO
P13796	P00387	0.261	0.809	0.529
O95865	P61026	0.265	0.797	0.536
P13796	Q12907	0.108	0.845	0.631
O95865	P11233	0.257	0.833	0.491
Q8TEB7	P51148	0.112	0.783	0.685
O95865	Q12907	0.065	0.819	0.694
Q8TEB7	P61586	0.182	0.876	0.516
Q13564	P61006	0.371	0.697	0.503
Q92560	Q13724	0.426	0.723	0.419
P13796	P61019	0.261	0.788	0.516
P62258	P61019	0.514	0.856	0.187
P49703	O95573	0.291	0.737	0.525
Q8TEB7	O00116	0.221	0.781	0.550
Q8TEB7	Q9BQE4	0.491	0.787	0.262
Q8TEB7	P61026	0.105	0.845	0.579
P62258	Q9NP72	0.528	0.803	0.187
Q8TEB7	Q9H7Z7	0.230	0.846	0.426
P62258	Q9H7Z7	0.379	0.861	0.262
P49703	O43169		0.816	0.685
O95865	Q8WUY8	0.637	0.682	0.182
P49703	P63218	0.038	0.775	0.685
O95865	Q9BQE4	0.444	0.862	0.191
O95865	O43169		0.803	0.694
P62258	P51148	0.471	0.837	0.187
Q92560	Q9H7Z7	0.485	0.783	0.200
P62258	Q96DA6	0.576	0.794	0.093
O95865	Q8NBX0	0.421	0.812	0.221
P49703	Q5VT66	0.044	0.816	0.590
Q92560	Q6ZRP7	0.389	0.785	0.271
P62258	Q12907	0.340	0.861	0.242
P49703	Q96DA6	0.550	0.777	0.113
P49703	P21964	0.110	0.797	0.530
P49703	Q9BQE4	0.505	0.746	0.183
P62258	Q5JTV8	0.223	0.683	0.520
P13796	Q9H7Z7	0.267	0.760	0.385
Q8TEB7	Q13724	0.535	0.793	0.083
Q13564	P63218	0.458	0.819	0.128
P62258	P00387	0.315	0.862	0.202
Q8TEB7	P11233	0.105	0.750	0.516
Q53GL0	P11233	0.547	0.822	
Q92560	Q9BQE4	0.573	0.746	0.049
Q13564	P61026	0.317	0.761	0.283
Q13564	P61019	0.482	0.656	0.221
P13796	Q5VT66	0.105	0.724	0.529
P49703	P62873	0.069	0.729	0.557

SARS-CoV-1	SARS-CoV-2	BPO	CCO	MFO
P13796	O43169		0.724	0.631
Q92560	P51149	0.457	0.730	0.167
O95865	P62873	0.291	0.817	0.241
Q92560	Q8N183	0.278	0.798	0.271
P62258	O00116	0.379	0.785	0.171
Q92560	P00387	0.353	0.771	0.209
O95865	P63218	0.347	0.821	0.158
Q13564	Q9NP72	0.286	0.806	0.221
P62258	P21964	0.251	0.870	0.190
P13796	O00116	0.137	0.714	0.454
Q13564	Q5JTV8	0.128	0.638	0.533
O95865	Q8WVC6	0.735		0.559
P13796	P63218	0.300	0.951	0.039
Q53GL0	P61586	0.492	0.796	
Q9HCD5	Q9H7Z7	0.280	0.764	0.241
P62258	Q8NBX0	0.298	0.898	0.077
Q13564	Q13724	0.402	0.667	0.197
Q13564	Q96A26	0.470	0.795	
Q92560	O00116	0.211	0.855	0.197
Q9HCD5	Q8WUY8	0.077	0.816	0.364
Q92560	Q8NBX0	0.221	0.763	0.271
Q13564	P61106	0.281	0.691	0.283
Q9HCD5	Q9BQE4	0.301	0.844	0.102
Q53GL0	P61026	0.522	0.725	
Q92560	P21964	0.381	0.757	0.108
Q92560	Q9NP72	0.305	0.772	0.167
O95865	Q8N183	0.220	0.803	0.221
A9UHW6	Q8WUY8		0.875	0.364
P13796	Q96DA6	0.334	0.785	0.116
Q53GL0	Q9NP72	0.387	0.843	
O95865	Q7LGA3	0.473	0.754	
Q92560	P61019	0.302	0.754	0.167
Q92560	P62820	0.334	0.720	0.167
Q53GL0	P62820	0.509	0.710	
Q53GL0	P61006	0.489	0.730	
Q13564	P00387	0.155	0.736	0.325
Q13564	P51148	0.307	0.679	0.221
Q92560	P61006	0.400	0.685	0.120
Q13564	P62820	0.307	0.677	0.221
Q8TEB7	Q7LGA3	0.339	0.865	
Q92560	P61586	0.332	0.750	0.120
Q92560	P62873	0.303	0.769	0.129
Q13564	P51149	0.231	0.749	0.221
Q13564	P21964	0.167	0.794	0.240
P13796	Q8N183	0.375	0.724	0.101

SARS-CoV-1	SARS-CoV-2	BPO	CCO	MFO
Q92560	Q96DA6	0.230	0.862	0.101
Q9HCD5	Q8WTV0	0.200	0.788	0.200
Q13564	Q5VT66	0.118	0.745	0.325
Q13564	Q8WTV0	0.256	0.704	0.227
Q92560	Q8WUY8	0.233	0.830	0.120
Q8TEB7	Q8WUY8	0.308	0.709	0.161
Q92560	P61106	0.353	0.682	0.138
Q13564	Q8N183	0.140	0.745	0.286
Q13564	O00116	0.126	0.721	0.314
Q53GL0	Q96DA6	0.346	0.814	
Q92560	O95573	0.265	0.723	0.169
Q92560	P61026	0.273	0.744	0.138
Q92560	Q5VT66	0.143	0.798	0.209
Q92560	Q8WTV0	0.380	0.683	0.083
Q92560	P51148	0.315	0.663	0.167
Q13564	Q9H7Z7	0.147	0.683	0.311
Q8TEB7	Q8NBX0	0.245	0.771	0.119
Q13564	Q8NBX0	0.128	0.721	0.286
P62258	Q8N183	0.309	0.744	0.077
Q53GL0	Q8WTV0	0.342	0.783	
P13796	Q96A26	0.361	0.763	
P62258	Q8WUY8	0.119	0.692	0.302
Q53GL0	P62873	0.354	0.751	
P62258	P63218	0.197	0.887	0.019
P62258	Q13724	0.248	0.807	0.044
O95865	Q6ZRP7	0.076	0.786	0.233
P49703	Q5JTV8	0.056	0.839	0.199
P13796	Q13724	0.183	0.843	0.066
Q53GL0	O95573	0.329	0.758	
P62258	Q6ZRP7	0.162	0.844	0.078
Q8TEB7	Q8N183	0.160	0.804	0.119
Q13564	Q96DA6	0.135	0.824	0.123
Q9HCD5	P21964	0.106	0.834	0.141
Q92560	Q5JTV8	0.198	0.807	0.075
P49703	Q8WUY8	0.071	0.867	0.142
Q92560	P63218	0.085	0.778	0.210
Q53GL0	Q96A26	0.224	0.842	
Q9HCD5	P62873	0.229	0.756	0.080
A9UHW6	O00116		0.936	0.125
Q92560	P11233	0.231	0.705	0.120
Q9HCD5	P51149	0.192	0.719	0.138
Q13564	Q6ZRP7	0.063	0.675	0.311
P13796	Q8NBX0	0.139	0.808	0.101
Q53GL0	Q5JTV8	0.177	0.870	
A9UHW6	Q9H7Z7		0.806	0.241

SARS-CoV-1	SARS-CoV-2	BPO	CCO	MFO
P62258	Q96A26	0.268	0.776	
Q92560	Q7LGA3	0.247	0.795	
Q9HCD5	P61586	0.072	0.845	0.124
Q8TEB7	P62873	0.101	0.730	0.210
Q53GL0	Q8WUY8	0.134	0.905	
Q9HCD5	Q12907	0.050	0.788	0.200
O95865	Q96DA6	0.144	0.772	0.122
Q53GL0	Q8N183	0.175	0.860	
P49703	Q6ZRP7	0.050	0.820	0.165
A9UHW6	O43169		0.834	0.200
Q9HCD5	P11233	0.082	0.827	0.124
P49703	Q8N183	0.050	0.816	0.160
P62258	Q5VT66	0.079	0.744	0.202
P13796	Q6ZRP7	0.101	0.816	0.105
Q53GL0	P21964	0.190	0.831	
Q8TEB7	Q96DA6	0.113	0.773	0.133
O95865	Q96A26	0.174	0.837	
Q8TEB7	Q5JTV8	0.086	0.690	0.230
Q53GL0	Q8NBX0	0.141	0.863	
Q9HCD5	P51148	0.097	0.763	0.138
Q53GL0	P51149	0.287	0.711	
Q9HCD5	P61019	0.062	0.797	0.138
Q13564	Q8WUY8	0.140	0.665	0.187
A9UHW6	Q5VT66		0.834	0.155
Q9HCD5	P62820	0.074	0.774	0.138
P62258	O43169		0.744	0.242
Q8TEB7	Q8WVC6	0.296		0.689
Q53GL0	Q7LGA3	0.125	0.856	
Q9HCD5	P61006	0.065	0.788	0.124
Q92560	Q96A26	0.137	0.838	
Q53GL0	O00116	0.138	0.836	
Q53GL0	Q5VT66	0.109	0.860	
O95865	Q5JTV8	0.103	0.661	0.204
Q9HCD5	Q13724	0.070	0.863	0.034
P49703	Q13724	0.068	0.732	0.167
Q13564	Q12907	0.043	0.703	0.215
Q13564	O43169		0.745	0.215
Q9HCD5	Q5JTV8	0.066	0.792	0.097
Q53GL0	Q9H7Z7	0.182	0.772	
P13796	Q8WUY8	0.140	0.666	0.138
A9UHW6	Q5JTV8		0.845	0.097
Q53GL0	P51148	0.190	0.749	
Q9HCD5	P61106	0.087	0.722	0.129
Q9HCD5	P61026	0.083	0.725	0.129
Q9HCD5	P63218	0.103	0.821	0.012

SARS-CoV-1	SARS-CoV-2	BPO	CCO	MFO
Q9HCD5	P00387	0.081	0.700	0.155
A9UHW6	Q9NP72		0.798	0.138
Q9HCD5	Q8NBX0	0.067	0.804	0.064
Q8TEB7	Q96A26	0.097	0.838	
Q53GL0	P63218	0.128	0.806	
Q53GL0	P61106	0.207	0.724	
Q53GL0	Q13724	0.167	0.763	
Q9HCD5	Q5VT66	0.053	0.719	0.155
Q53GL0	Q9BQE4	0.158	0.767	
A9UHW6	P21964		0.783	0.141
Q9HCD5	O43169		0.719	0.200
Q53GL0	Q6ZRP7	0.093	0.826	
Q8TEB7	Q6ZRP7	0.069	0.725	0.119
A9UHW6	P61019		0.774	0.138
Q9HCD5	O00116	0.058	0.721	0.125
A9UHW6	Q8WTV0		0.702	0.200
A9UHW6	Q12907		0.701	0.200
A9UHW6	Q8N183		0.834	0.064
A9UHW6	P61586		0.770	0.124
A9UHW6	O95573		0.751	0.142
Q53GL0	Q12907	0.111	0.781	
Q9HCD5	Q9NP72	0.097	0.651	0.138
Q13564	Q7LGA3	0.141	0.743	
P49703	Q7LGA3	0.072	0.812	
Q92560	O43169		0.798	0.083
A9UHW6	P00387		0.726	0.155
Q53GL0	P61019	0.138	0.741	
A9UHW6	P62820		0.740	0.138
P49703	Q96A26	0.072	0.803	
Q9HCD5	O95573	0.063	0.669	0.142
A9UHW6	Q96DA6		0.794	0.080
A9UHW6	Q96A26		0.874	
Q53GL0	P00387	0.179	0.694	
Q92560	Q12907	0.102	0.682	0.083
Q53GL0	O43169		0.860	
A9UHW6	P11233		0.728	0.124
A9UHW6	Q8NBX0		0.787	0.064
P13796	Q7LGA3	0.126	0.723	
Q9HCD5	Q96DA6	0.080	0.687	0.080
Q9HCD5	Q8N183	0.052	0.719	0.064
A9UHW6	Q6ZRP7		0.769	0.064
A9UHW6	Q7LGA3		0.830	
A9UHW6	P61006		0.704	0.124
A9UHW6	P61106		0.696	0.129
A9UHW6	P61026		0.696	0.129

SARS-CoV-1	SARS-CoV-2	BPO	CCO	MFO
A9UHW6	P51149		0.687	0.138
A9UHW6	P51148		0.683	0.138
P62258	Q7LGA3	0.113	0.702	
A9UHW6	P62873		0.718	0.080
Q8TEB7	P63218	0.058	0.685	0.054
A9UHW6	Q9BQE4		0.692	0.102
Q9HCD5	Q7LGA3	0.078	0.715	
A9UHW6	Q13724		0.748	0.034
Q9HCD5	Q96A26	0.078	0.703	
A9UHW6	P63218		0.713	0.012
P13796	Q8WVC6	0.147		0.518
P49703	Q8WVC6	0.066		0.530
Q13564	Q8WVC6	0.154		0.242
Q92560	Q8WVC6	0.243		0.106
P62258	Q8WVC6	0.129		0.190
Q9HCD5	Q8WVC6	0.067		0.141
Q53GL0	Q8WVC6	0.141		
A9UHW6	Q8WVC6			0.141

Nsp8

NP_828866 - - - - - - - - - - - - - - P0DTD1-PRO_0000449626

NP_828866:
P05155 P23588 P54274
P69849 Q9P0M6 Q9Y6E2

P0DTD1-PRO_0000449626:
O00566 O76094 O95260 O96028
P09132 P61011 P82663 P82675
Q13206 Q13868 Q4G0J3 Q7L2J0
Q8NEJ9 Q92552 Q96B26 Q96I59
Q9BSC4 Q9H6F5 Q9HD40 Q9NQT4
Q9NQT5 Q9NY61 Q9ULT8 Q9Y399

SARS-CoV-1	SARS-CoV-2	BPO	CCO	MFO
P23588	Q9HD40	0.619	0.827	0.917
P23588	Q92552	0.641	0.823	0.876
P23588	P09132	0.461	0.855	0.917
P23588	Q4G0J3	0.411	0.860	0.917
P23588	Q8NEJ9	0.477	0.767	0.917
P23588	Q9BSC4	0.477	0.750	0.917
P23588	P82675	0.699	0.823	0.591
P23588	O00566	0.477	0.701	0.917
P54274	O96028	0.485	0.895	0.618
P23588	Q13868	0.482	0.822	0.694
P23588	Q96B26	0.482	0.822	0.675

SARS-CoV-1	SARS-CoV-2	BPO	CCO	MFO
P23588	Q9NQT5	0.436	0.799	0.694
P23588	Q9NQT4	0.428	0.799	0.694
P23588	Q9Y399	0.950	0.823	0.121
P23588	O76094	0.054	0.899	0.917
P23588	P61011	0.428	0.822	0.593
Q9P0M6	P09132	0.517	0.785	0.528
Q9P0M6	O96028	0.498	0.826	0.463
P54274	Q9NY61	0.311	0.837	0.514
P23588	P82663	0.699	0.823	0.121
P23588	Q13206	0.438	0.691	0.496
P23588	Q9H6F5	0.052	0.655	0.917
P54274	Q9NQT5	0.435	0.949	0.239
P54274	Q9NQT4	0.426	0.949	0.239
Q9P0M6	O00566	0.196	0.884	0.528
Q9P0M6	P61011	0.473	0.816	0.317
P23588	Q96I59	0.619	0.768	0.164
Q9P0M6	Q8NEJ9	0.196	0.824	0.528
P54274	Q96B26	0.379	0.915	0.250
Q9P0M6	Q9BSC4	0.196	0.813	0.528
P54274	Q13868	0.379	0.915	0.239
P54274	P61011	0.343	0.915	0.268
P54274	O00566	0.328	0.942	0.255
P54274	Q9Y399	0.600	0.749	0.175
Q9P0M6	Q9NQT5	0.305	0.852	0.359
P54274	Q8NEJ9	0.328	0.921	0.255
P54274	P09132	0.355	0.891	0.255
P23588	Q9NY61	0.177	0.713	0.591
P54274	Q9HD40	0.430	0.789	0.255
P54274	Q96I59	0.430	0.824	0.219
P54274	Q9BSC4	0.328	0.876	0.255
P54274	Q92552	0.431	0.749	0.278
Q9P0M6	Q4G0J3	0.106	0.818	0.528
P54274	P82675	0.416	0.749	0.281
P54274	Q4G0J3	0.301	0.886	0.255
Q9P0M6	Q13206	0.442	0.736	0.256
Q9P0M6	Q9NY61	0.387	0.731	0.315
P23588	O96028	0.396	0.717	0.295
Q9P0M6	Q9NQT4	0.174	0.852	0.359
Q9P0M6	Q9H6F5	0.064	0.754	0.528
Q9P0M6	Q9HD40	0.100	0.714	0.528
Q9P0M6	Q13868	0.165	0.816	0.359
P54274	P82663	0.416	0.749	0.175
P54274	Q13206	0.327	0.758	0.246
Q9P0M6	Q96B26	0.165	0.816	0.349
Q9P0M6	Q9Y399	0.519	0.693	0.114

SARS-CoV-1	SARS-CoV-2	BPO	CCO	MFO
Q9P0M6	Q92552	0.110	0.693	0.485
Q9P0M6	O76094	0.063	0.694	0.528
Q9Y6E2	Q13206	0.478	0.772	
P05155	O76094	0.451	0.710	0.080
P05155	P61011	0.423	0.669	0.105
Q9Y6E2	Q9NY61	0.433	0.760	
P05155	P09132	0.394	0.686	0.080
P54274	Q9H6F5	0.070	0.825	0.255
P05155	Q9NQT4	0.408	0.658	0.083
P54274	O95260	0.185	0.797	0.162
P05155	Q9NY61	0.328	0.700	0.095
P05155	O96028	0.291	0.704	0.122
Q9P0M6	P82675	0.108	0.693	0.315
P23588	Q7L2J0	0.411		0.694
Q9Y6E2	O96028	0.325	0.765	
P54274	O76094	0.077	0.757	0.255
P23588	O95260	0.242	0.774	0.033
P05155	Q9NQT5	0.287	0.658	0.083
P05155	Q9Y399	0.123	0.696	0.190
Q9P0M6	Q96I59	0.100	0.752	0.155
P05155	P82663	0.120	0.696	0.190
P05155	P82675	0.120	0.696	0.163
Q9Y6E2	Q9NQT5	0.286	0.688	
P05155	O95260	0.121	0.745	0.101
P05155	Q96I59	0.127	0.702	0.134
P05155	Q9HD40	0.127	0.738	0.080
P05155	Q13206	0.159	0.682	0.098
Q9Y6E2	O95260	0.089	0.848	
P05155	Q92552	0.152	0.696	0.079
Q9Y6E2	Q9HD40	0.091	0.826	
Q9P0M6	P82663	0.108	0.693	0.114
P69849	O76094		0.913	
P69849	O95260		0.898	
Q9Y6E2	Q92552	0.130	0.753	
P69849	Q9HD40		0.876	
P05155	Q13868	0.123	0.669	0.083
P05155	Q96B26	0.123	0.669	0.078
P69849	Q9NY61		0.854	
Q9P0M6	O95260	0.090	0.728	0.034
Q9Y6E2	Q96I59	0.091	0.757	
Q9Y6E2	P82675	0.093	0.753	
Q9Y6E2	P82663	0.093	0.753	
P05155	Q4G0J3	0.076	0.690	0.080
P69849	Q13206		0.845	
Q9Y6E2	O76094	0.066	0.774	

SARS-CoV-1	SARS-CoV-2	BPO	CCO	MFO
Q9Y6E2	Q9Y399	0.086	0.753	
P05155	Q8NEJ9	0.088	0.665	0.080
P69849	Q9Y399		0.832	
P69849	Q92552		0.832	
P69849	P82675		0.832	
P69849	P82663		0.832	
P69849	Q96I59		0.830	
Q9Y6E2	Q4G0J3	0.087	0.740	
P05155	Q9H6F5	0.090	0.655	0.080
P69849	O96028		0.821	
Q9Y6E2	P09132	0.080	0.734	
Q9Y6E2	Q9NQT4	0.117	0.688	
P05155	Q9BSC4	0.088	0.631	0.080
P69849	Q4G0J3		0.795	
Q9Y6E2	Q96B26	0.087	0.706	
Q9Y6E2	Q13868	0.087	0.706	
P69849	Q9H6F5		0.793	
P69849	P09132		0.790	
Q9Y6E2	Q9H6F5	0.071	0.717	
Q9Y6E2	Q8NEJ9	0.086	0.700	
Q9Y6E2	P61011	0.080	0.706	
P69849	Q8NEJ9		0.776	
P05155	O00566	0.088	0.606	0.080
P69849	Q96B26		0.762	
P69849	Q13868		0.762	
P69849	P61011		0.762	
Q9Y6E2	Q9BSC4	0.086	0.671	
P69849	Q9BSC4		0.749	
P69849	Q9NQT5		0.743	
P69849	Q9NQT4		0.743	
Q9Y6E2	O00566	0.086	0.628	
P69849	O00566		0.701	
Q9P0M6	Q9ULT8	0.383		0.200
P54274	Q7L2J0	0.301		0.279
Q9Y6E2	Q9ULT8	0.565		
Q9P0M6	Q7L2J0	0.106		0.360
P23588	Q9ULT8	0.170		0.197
P54274	Q9ULT8	0.148		0.187
P05155	Q9ULT8	0.155		0.133
P05155	Q7L2J0	0.076		0.082
Q9Y6E2	Q7L2J0	0.087		

Nsp9

NP_828867 - - - - - - - - - - - - - P0DTD1-PRO_0000449627

P06733 Q5SQN1 Q6P587
Q7Z494 Q9BUV0 Q9UQN3

P13984 P35555 P35556 P35658
P37198 P61962 Q15056 Q7Z3B4
Q86YT6 Q8N0X7 Q8TD19 Q96F45
Q99567 Q9BVL2 Q9NZL9 Q9UBX5

SARS-CoV-1	SARS-CoV-2	BPO	CCO	MFO
P06733	Q9NZL9	0.651	0.970	0.364
P06733	P13984	0.547	0.769	0.548
P06733	Q15056	0.434	0.781	0.467
Q6P587	Q96F45	0.119	0.848	0.706
Q6P587	Q86YT6	0.099	0.777	0.706
Q6P587	Q8TD19	0.161	0.871	0.541
Q6P587	P13984	0.255	0.828	0.457
P06733	Q8TD19	0.176	0.785	0.554
P06733	P37198	0.309	0.789	0.414
Q7Z494	Q86YT6	0.695	0.795	
Q5SQN1	Q9BVL2	0.713	0.772	
Q5SQN1	Q7Z3B4	0.709	0.772	
Q7Z494	P35555	0.589	0.856	
Q6P587	P35555	0.118	0.718	0.605
P06733	P35555	0.158	0.831	0.415
Q6P587	Q9NZL9	0.368	0.815	0.198
Q9UQN3	P37198	0.676	0.697	
Q6P587	P37198	0.148	0.789	0.426
Q9UQN3	P35658	0.609	0.736	
Q9UQN3	Q99567	0.605	0.736	
P06733	Q86YT6	0.145	0.774	0.418
Q5SQN1	Q86YT6	0.429	0.894	
Q5SQN1	P37198	0.538	0.784	
Q5SQN1	Q99567	0.484	0.834	
P06733	Q96F45	0.152	0.741	0.418
Q5SQN1	P35658	0.474	0.834	
P06733	Q8N0X7	0.122	0.795	0.380
Q5SQN1	Q15056	0.395	0.866	
Q9UQN3	Q7Z3B4	0.558	0.670	
Q9UQN3	Q9BVL2	0.554	0.670	
Q6P587	P35658	0.102	0.903	0.210
Q6P587	Q99567	0.096	0.903	0.210
Q7Z494	P37198	0.473	0.722	
Q7Z494	Q8N0X7	0.370	0.818	

SARS-CoV-1	SARS-CoV-2	BPO	CCO	MFO
Q9UQN3	Q8TD19	0.490	0.697	
P06733	P35658	0.147	0.841	0.188
Q6P587	Q8N0X7	0.108	0.863	0.203
P06733	Q99567	0.143	0.841	0.188
Q7Z494	Q9BVL2	0.449	0.704	
Q7Z494	Q7Z3B4	0.449	0.704	
Q5SQN1	Q8TD19	0.352	0.789	
Q7Z494	P61962	0.332	0.803	
Q9UQN3	Q86YT6	0.352	0.780	
Q6P587	Q15056	0.195	0.768	0.164
P06733	Q9UBX5	0.121	0.591	0.415
Q7Z494	Q99567	0.343	0.776	
Q9UQN3	Q8N0X7	0.349	0.768	
P06733	Q9BVL2	0.141	0.787	0.188
Q9UQN3	Q15056	0.376	0.734	
Q7Z494	P35658	0.333	0.776	
P06733	Q7Z3B4	0.134	0.787	0.188
Q6P587	Q9UBX5	0.109	0.385	0.605
Q6P587	Q9BVL2	0.101	0.786	0.210
Q7Z494	Q15056	0.309	0.781	
Q6P587	Q7Z3B4	0.092	0.786	0.210
Q6P587	P61962	0.162	0.926	
Q7Z494	Q9UBX5	0.474	0.591	
Q7Z494	Q8TD19	0.231	0.816	
P06733	P61962	0.180	0.859	
Q9UQN3	Q9NZL9	0.170	0.861	
Q7Z494	P13984	0.332	0.689	
Q5SQN1	Q8N0X7	0.147	0.863	
Q9UQN3	Q9UBX5	0.460	0.543	
Q5SQN1	P13984	0.249	0.754	
Q5SQN1	Q9UBX5	0.649	0.353	
Q9UQN3	P35555	0.199	0.802	
Q7Z494	Q9NZL9	0.121	0.869	
Q7Z494	Q96F45	0.205	0.771	
Q7Z494	P35556	0.543	0.429	
Q9UQN3	P13984	0.284	0.662	
Q5SQN1	P61962	0.080	0.864	
Q5SQN1	Q9NZL9	0.118	0.820	
Q5SQN1	P35555	0.235	0.692	
P06733	P35556	0.109	0.403	0.415
Q9UQN3	Q96F45	0.294	0.628	
Q5SQN1	Q96F45	0.166	0.729	
Q6P587	P35556	0.082	0.195	0.605
Q9UQN3	P61962	0.094	0.754	
Q9UQN3	P35556	0.195	0.343	

SARS-CoV-1	SARS-CoV-2	BPO	CCO	MFO
Q5SQN1	P35556	0.190	0.214	

Nsp10

NP_828868 - - - - - - P0DTD1-PRO_0000449628

P20290 P35354 Q9Y2D1

O94973 P55789 Q969X5 Q96CW1 Q9HAV7

SARS-CoV-1	SARS-CoV-2	BPO	CCO	MFO
P35354	P55789	0.215	0.782	0.852
P35354	O94973	0.305	0.862	0.575
P20290	Q9HAV7	0.524	0.906	0.095
Q9Y2D1	O94973	0.200	0.772	0.501
P20290	O94973	0.451	0.808	0.168
P35354	Q96CW1	0.292	0.801	0.284
P20290	Q96CW1	0.411	0.755	0.200
P35354	Q9HAV7	0.147	0.870	0.265
Q9Y2D1	Q9HAV7	0.155	0.858	0.267
P20290	Q969X5	0.439	0.806	
Q9Y2D1	Q96CW1	0.192	0.727	0.226
Q9Y2D1	P55789	0.136	0.760	0.213
P20290	P55789	0.066	0.804	0.155
Q9Y2D1	Q969X5	0.117	0.824	
P35354	Q969X5	0.082	0.822	

Nsp12

NP_828869 - - - - - - P0DTD1-PRO_0000449629

O95299 P09630 P20231 P30876 P51948 Q15661 Q5SQN1 Q8TD31 Q99426 Q99471 Q9UQN3

A3KN83 O14874 O43823 O75592 O95391 Q13546 Q14157 Q2T9J0 Q5EBL8 Q5JSZ5 Q5T6F2 Q5VUA4 Q6UUV7 Q70EL1 Q8IWR0 Q92615 Q96IZ5 Q99081 Q9H2H8 Q9HAU0

SARS-CoV-1	SARS-CoV-2	BPO	CCO	MFO
P09630	Q99081	0.586	0.878	0.823

SARS-CoV-1	SARS-CoV-2	BPO	CCO	MFO
P30876	O95391	0.496	0.889	0.766
P30876	Q8IWR0	0.474	0.816	0.766
P30876	Q96IZ5	0.618	0.884	0.550
P51948	Q13546	0.578	0.825	0.636
P09630	Q9HAU0	0.835	1.000	0.186
P51948	O95391	0.607	1.000	0.409
P30876	O43823	0.379	0.852	0.732
P51948	O14874	0.330	0.862	0.698
Q99471	Q9HAU0	0.708	0.883	0.262
P51948	Q5VUA4	0.327	0.954	0.451
P51948	O75592	0.420	0.854	0.420
Q99471	Q99081	0.527	0.899	0.252
Q99471	O75592	0.406	0.898	0.352
P30876	Q13546	0.440	0.701	0.512
P51948	O43823	0.442	0.837	0.353
P51948	Q9HAU0	0.230	0.954	0.439
P51948	Q9H2H8	0.548	0.897	0.175
Q99426	Q9HAU0	0.739	0.873	
Q5SQN1	Q2T9J0	0.772	0.837	
P30876	Q14157	0.144	0.914	0.550
P30876	O14874	0.279	0.773	0.554
P30876	Q99081	0.156	0.978	0.471
Q99426	Q99081	0.842	0.752	
P30876	Q9H2H8	0.561	0.884	0.138
P51948	Q8IWR0	0.328	0.830	0.409
O95299	Q9H2H8	0.273	0.877	0.387
Q5SQN1	Q5EBL8	0.660	0.876	
Q5SQN1	O95391	0.672	0.864	
O95299	Q2T9J0	0.473	0.838	0.209
Q99426	O75592	0.592	0.927	
P30876	Q5VUA4	0.182	0.835	0.494
P09630	O75592	0.438	0.896	0.164
Q99471	Q9H2H8	0.439	0.965	0.083
O95299	Q13546	0.434	0.883	0.161
P09630	Q14157	0.160	0.925	0.383
Q99471	Q6UUV7	0.183	0.883	0.397
P51948	Q6UUV7	0.358	0.954	0.136
O95299	O95391	0.479	0.862	0.101
P30876	O75592	0.192	0.791	0.455
P51948	Q96IZ5	0.428	0.897	0.103
P09630	O43823	0.112	0.868	0.443
O95299	Q6UUV7	0.533	0.798	0.083
Q8TD31	O75592	0.466	0.945	
P51948	Q99081	0.296	0.926	0.175
P30876	Q9HAU0	0.085	0.835	0.474

SARS-CoV-1	SARS-CoV-2	BPO	CCO	MFO
Q99471	Q5VUA4	0.237	0.883	0.262
P51948	Q2T9J0	0.357	0.831	0.194
P09630	O95391	0.076	0.954	0.350
P30876	Q92615		0.828	0.550
Q8TD31	Q99081	0.575	0.796	
Q5SQN1	Q13546	0.424	0.943	
O95299	O14874	0.168	1.000	0.187
Q8TD31	Q2T9J0	0.509	0.845	
Q9UQN3	Q13546	0.466	0.887	
P30876	Q5T6F2		0.801	0.550
P09630	Q5VUA4	0.161	1.000	0.182
Q8TD31	O95391	0.479	0.858	
P09630	Q5T6F2		0.931	0.383
Q9UQN3	O43823	0.651	0.659	
Q9UQN3	Q5EBL8	0.463	0.842	
Q99471	O95391	0.150	0.941	0.209
Q8TD31	Q9HAU0	0.404	0.895	
P09630	Q8IWR0	0.060	0.878	0.350
Q99471	Q8IWR0	0.183	0.893	0.209
Q99471	Q13546	0.185	0.820	0.273
P51948	Q5EBL8	0.483	0.794	
P09630	Q96IZ5	0.056	0.830	0.383
Q99471	O43823	0.192	0.800	0.276
O95299	Q5EBL8	0.481	0.784	
P09630	Q92615		0.874	0.383
Q5SQN1	Q6UUV7	0.445	0.809	
Q8TD31	O43823	0.460	0.791	
Q99471	Q14157	0.179	0.964	0.102
Q99471	Q96IZ5	0.172	0.965	0.102
Q99471	Q2T9J0	0.130	0.825	0.281
P09630	Q6UUV7	0.126	1.000	0.109
Q99471	O14874	0.128	0.919	0.186
Q9UQN3	Q2T9J0	0.502	0.727	
Q9UQN3	O95391	0.474	0.754	
P51948	Q14157	0.147	0.977	0.103
Q5SQN1	O43823	0.520	0.698	
O95299	Q96IZ5	0.229	0.877	0.064
Q9UQN3	Q6UUV7	0.456	0.707	
Q99426	Q5VUA4	0.261	0.873	
O95299	Q8IWR0	0.236	0.795	0.101
P30876	Q6UUV7	0.133	0.835	0.153
O95299	O75592	0.187	0.817	0.107
Q8TD31	Q5VUA4	0.205	0.895	
O95299	Q14157	0.134	0.879	0.064
O95299	O43823	0.196	0.791	0.089

SARS-CoV-1	SARS-CoV-2	BPO	CCO	MFO
O95299	Q5VUA4	0.148	0.798	0.119
Q8TD31	Q5EBL8	0.343	0.715	
O95299	Q99081	0.133	0.814	0.109
Q15661	Q2T9J0	0.162	0.210	0.683
P09630	Q2T9J0	0.068	0.872	0.111
P09630	O14874	0.091	0.798	0.161
Q15661	Q70EL1	0.048		1.000
P30876	Q5EBL8	0.386	0.656	
Q99471	Q5T6F2		0.937	0.102
P09630	Q13546	0.114	0.774	0.151
Q9UQN3	Q5VUA4	0.327	0.707	
P09630	Q9H2H8	0.080	0.830	0.118
P51948	Q92615		0.922	0.103
Q8TD31	Q6UUV7	0.129	0.895	
Q99471	Q92615		0.904	0.102
Q99426	Q6UUV7	0.129	0.873	
P20231	Q70EL1			1.000
O95299	Q9HAU0	0.080	0.798	0.119
Q5SQN1	Q5VUA4	0.184	0.809	
Q99426	Q2T9J0	0.078	0.911	
P51948	Q5T6F2		0.883	0.103
Q9UQN3	Q14157	0.256	0.724	
P30876	Q2T9J0	0.139	0.680	0.161
Q99471	Q5EBL8	0.195	0.783	
Q8TD31	Q14157	0.148	0.825	
Q9UQN3	O14874	0.213	0.759	
Q9UQN3	O75592	0.293	0.677	
Q5SQN1	Q14157	0.137	0.831	
Q5SQN1	O75592	0.186	0.776	
Q5SQN1	O14874	0.094	0.864	
Q99426	Q13546	0.161	0.795	
Q5SQN1	Q9H2H8	0.152	0.803	
Q99426	Q14157	0.161	0.792	
Q99426	O43823	0.189	0.753	
Q5SQN1	Q99081	0.154	0.780	
Q99426	O14874	0.099	0.827	
Q99426	O95391	0.083	0.832	
Q8TD31	Q13546	0.154	0.759	
O95299	Q5T6F2		0.849	0.064
Q8TD31	Q9H2H8	0.103	0.795	
Q8TD31	Q8IWR0	0.061	0.835	
Q99426	Q8IWR0	0.087	0.808	
Q99471	A3KN83		0.893	
P20231	Q2T9J0		0.210	0.683
O95299	Q92615		0.829	0.064

SARS-CoV-1	SARS-CoV-2	BPO	CCO	MFO
Q9UQN3	Q99081	0.197	0.694	
Q5SQN1	Q96IZ5	0.088	0.803	
Q5SQN1	Q9HAU0	0.081	0.809	
Q8TD31	Q5T6F2		0.888	
Q99426	Q5EBL8	0.130	0.756	
Q99426	Q9H2H8	0.121	0.763	
Q9UQN3	Q9H2H8	0.194	0.687	
P09630	A3KN83		0.878	
Q99426	Q5T6F2		0.873	
Q8TD31	O14874	0.088	0.777	
Q8TD31	Q96IZ5	0.058	0.795	
Q99426	Q96IZ5	0.076	0.763	
P09630	Q5EBL8	0.109	0.730	
Q8TD31	A3KN83		0.835	
Q5SQN1	Q92615		0.834	
Q8TD31	Q92615		0.832	
P51948	A3KN83		0.830	
Q5SQN1	Q8IWR0	0.090	0.729	
P30876	A3KN83		0.816	
Q99426	Q92615		0.808	
Q99426	A3KN83		0.808	
Q9UQN3	Q96IZ5	0.112	0.687	
Q5SQN1	Q5T6F2		0.799	
Q9UQN3	Q9HAU0	0.090	0.707	
O95299	A3KN83		0.795	
Q9UQN3	Q8IWR0	0.111	0.628	
Q9UQN3	Q92615		0.736	
Q5SQN1	A3KN83		0.729	
Q9UQN3	Q5T6F2		0.688	
Q15661	Q13546	0.358	0.197	0.092
Q15661	Q9H2H8	0.119	0.242	0.271
Q9UQN3	A3KN83		0.628	
Q15661	Q5EBL8	0.183	0.439	
Q15661	O43823	0.364	0.180	0.055
Q15661	O95391	0.317	0.212	0.066
P30876	Q5JSZ5			0.550
Q15661	Q5VUA4	0.263	0.203	0.083
P20231	Q9H2H8		0.242	0.271
Q15661	O75592	0.197	0.213	0.071
Q15661	Q6UUV7	0.221	0.203	0.049
Q15661	Q99081	0.193	0.202	0.077
Q15661	O14874	0.112	0.230	0.106
P20231	Q5EBL8		0.439	
Q15661	Q14157	0.157	0.220	0.034
P51948	Q70EL1	0.259		0.144

SARS-CoV-1	SARS-CoV-2	BPO	CCO	MFO
Q15661	Q8IWR0	0.093	0.233	0.066
Q15661	Q9HAU0	0.105	0.203	0.083
P09630	Q5JSZ5			0.383
O95299	Q70EL1	0.110		0.271
Q15661	Q96IZ5	0.081	0.242	0.034
P20231	O14874		0.230	0.106
P20231	Q8IWR0		0.233	0.066
P20231	Q13546		0.197	0.092
P20231	Q9HAU0		0.203	0.083
P20231	Q5VUA4		0.203	0.083
P20231	O75592		0.213	0.071
P20231	Q99081		0.202	0.077
P20231	O95391		0.212	0.066
P20231	Q96IZ5		0.242	0.034
Q15661	Q5T6F2		0.223	0.034
P20231	Q5T6F2		0.223	0.034
P20231	Q14157		0.220	0.034
P20231	Q6UUV7		0.203	0.049
P30876	Q70EL1	0.173		0.077
Q15661	Q92615		0.201	0.034
P20231	Q92615		0.201	0.034
P20231	O43823		0.180	0.055
Q15661	A3KN83		0.233	
P20231	A3KN83		0.233	
Q99471	Q70EL1	0.105		0.049
P09630	Q70EL1	0.035		0.087
P51948	Q5JSZ5			0.103
Q99471	Q5JSZ5			0.102
O95299	Q5JSZ5			0.064
Q9UQN3	Q70EL1	0.057		
Q5SQN1	Q70EL1	0.045		
Q99426	Q70EL1	0.042		
Q15661	Q5JSZ5			0.034
P20231	Q5JSZ5			0.034
Q8TD31	Q70EL1	0.030		

Nsp15

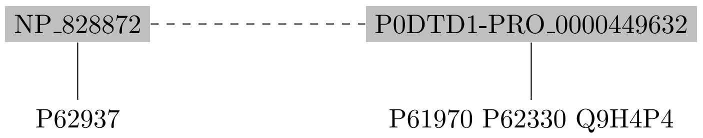

SARS-CoV-1	SARS-CoV-2	BPO	CCO	MFO
P62937	P62330	0.378	0.867	0.269
P62937	P61970	0.293	0.932	0.270
P62937	Q9H4P4	0.424	0.770	0.275

C Accessory Proteins

Orf3a

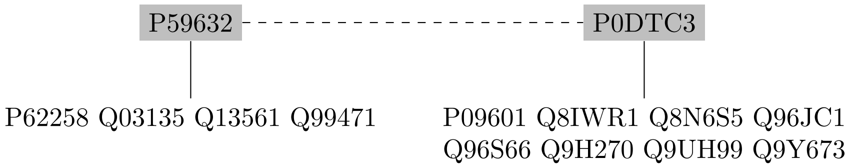

SARS-CoV-1	SARS-CoV-2	BPO	CCO	MFO
Q13561	Q9UH99	0.625	0.751	0.688
Q03135	P09601	0.697	0.750	0.458
P62258	P09601	0.519	0.877	0.509
Q03135	Q9UH99	0.614	0.809	0.475
P62258	Q9UH99	0.720	0.782	0.317
Q13561	P09601	0.467	0.794	0.548
Q03135	Q9H270	0.424	0.855	0.494
Q99471	Q9UH99	0.326	0.852	0.383
Q13561	Q9H270	0.501	0.725	0.332
P62258	Q9H270	0.483	0.767	0.294
Q03135	Q8IWR1	0.318	0.851	0.268
Q99471	Q9Y673	0.479	0.845	0.049
Q99471	P09601	0.317	0.757	0.285
Q99471	Q8IWR1	0.177	0.915	0.262
Q99471	Q9H270	0.180	0.833	0.325
Q13561	Q8IWR1	0.302	0.764	0.262
P62258	Q8IWR1	0.328	0.754	0.242
Q03135	Q96JC1	0.424	0.855	
P62258	Q96JC1	0.483	0.767	
Q13561	Q96JC1	0.501	0.725	
Q03135	Q96S66	0.222	0.855	0.140
Q13561	Q96S66	0.366	0.734	0.085
P62258	Q96S66	0.298	0.784	0.078
Q03135	Q9Y673	0.226	0.801	0.104
Q99471	Q96S66	0.087	0.927	0.085
P62258	Q9Y673	0.275	0.702	0.044
Q99471	Q96JC1	0.180	0.833	
Q13561	Q9Y673	0.120	0.706	0.049

Orf6

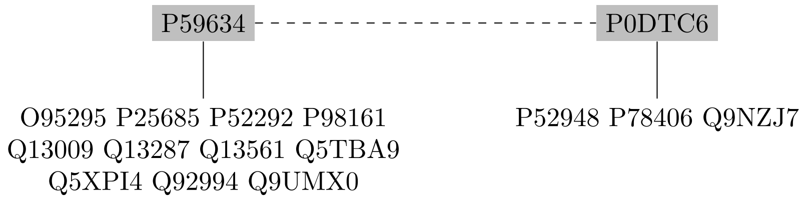

SARS-CoV-1	SARS-CoV-2	BPO	CCO	MFO
P52292	P78406	0.631	0.802	0.638
P52292	P52948	0.566	0.874	0.499
Q13561	P78406	0.541	0.778	0.579
Q92994	P52948	0.545	0.883	0.292
Q13009	P78406	0.232	0.847	0.543
P98161	P78406	0.562	0.750	0.276
Q5TBA9	Q9NZJ7	0.668	0.854	
P98161	P52948	0.583	0.747	0.141
Q92994	P78406	0.171	0.858	0.418
Q13561	P52948	0.476	0.796	0.121
Q5XPI4	P78406	0.267	0.870	0.247
Q13009	Q9NZJ7	0.562	0.814	
Q5XPI4	P52948	0.301	0.901	0.170
Q9UMX0	P52948	0.359	0.862	0.112
Q9UMX0	P78406	0.182	0.803	0.323
O95295	P52948	0.503	0.790	
O95295	P78406	0.508	0.758	
O95295	Q9NZJ7	0.471	0.793	
P25685	P78406	0.149	0.794	0.298
Q13287	P52948	0.275	0.900	
Q13009	P52948	0.169	0.829	0.170
P25685	Q9NZJ7	0.427	0.727	
P25685	P52948	0.143	0.822	0.149
Q5TBA9	P78406	0.167	0.810	0.095
P98161	Q9NZJ7	0.326	0.707	
Q13287	Q9NZJ7	0.182	0.839	
Q5TBA9	P52948	0.146	0.734	0.135
Q13561	Q9NZJ7	0.307	0.708	
Q13287	P78406	0.174	0.825	
Q9UMX0	Q9NZJ7	0.241	0.751	
Q92994	Q9NZJ7	0.226	0.759	
P52292	Q9NZJ7	0.152	0.808	
Q5XPI4	Q9NZJ7	0.092	0.836	

Orf7a

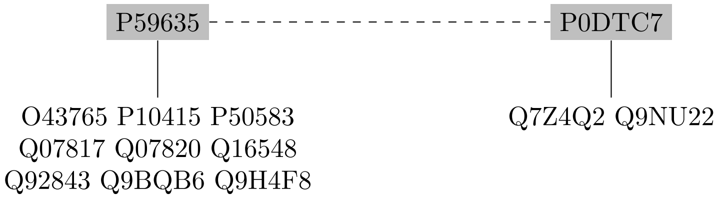

SARS-CoV-1	SARS-CoV-2	BPO	CCO	MFO
P50583	Q9NU22	0.327	0.777	0.579
P10415	Q9NU22	0.266	0.847	0.262
Q07817	Q9NU22	0.174	0.817	0.270
Q07817	Q7Z4Q2	0.264	0.464	0.397
Q07820	Q9NU22	0.089	0.884	0.096
O43765	Q9NU22	0.212	0.842	
Q07820	Q7Z4Q2	0.439	0.517	0.085
Q9BQB6	Q9NU22	0.108	0.774	0.141
O43765	Q7Z4Q2	0.489	0.531	
P10415	Q7Z4Q2	0.237	0.482	0.276
P50583	Q7Z4Q2	0.067	0.616	0.262
Q92843	Q9NU22	0.119	0.783	
Q16548	Q9NU22	0.099	0.777	
Q9H4F8	Q9NU22	0.083	0.187	0.579
Q9BQB6	Q7Z4Q2	0.069	0.612	0.083
Q16548	Q7Z4Q2	0.069	0.616	
Q92843	Q7Z4Q2	0.107	0.554	
Q9H4F8	Q7Z4Q2	0.070	0.266	0.262

Orf9b

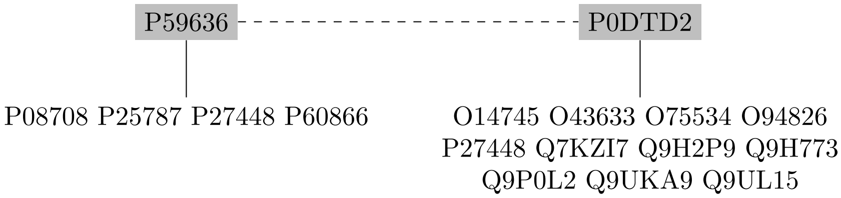

SARS-CoV-1	SARS-CoV-2	BPO	CCO	MFO
P27448	Q9P0L2	0.647	0.830	0.911
P27448	Q7KZI7	0.569	0.765	0.746
P27448	O14745	0.397	0.898	0.539

SARS-CoV-1	SARS-CoV-2	BPO	CCO	MFO
P27448	Q9H773	0.218	0.794	0.582
P27448	O43633	0.214	0.812	0.542
P27448	Q9H2P9	0.456	0.770	0.130
P27448	Q9UL15	0.193	0.794	0.273
P27448	O94826	0.178	0.868	0.099
P27448	O75534	0.142	0.831	0.131
P27448	Q9UKA9	0.206	0.681	0.135
